# Dynamics of Ligand and Guest in 1D Hg(II)‐Bispidine Coordination Polymers With Different Topologies Investigated by Solid‐State NMR

**DOI:** 10.1002/chem.202501458

**Published:** 2025-07-14

**Authors:** Elisa Della Latta, Francesco Della Croce, Giuditta Bizai, Andrea Murelli, Martina Lippi, Patrizia Rossi, Massimo Cametti, Paola Paoli, Francesca Martini, Lucia Calucci, Marco Geppi

**Affiliations:** ^1^ Dipartimento di Chimica e Chimica Industriale Università di Pisa via G. Moruzzi 13 Pisa 56124 Italy; ^2^ Istituto di Chimica dei Composti OrganoMetallici – ICCOM Consiglio Nazionale delle Ricerche – CNR via G. Moruzzi 1 Pisa 56124 Italy; ^3^ Dipartimento di Chimica, Materiali e Ingegneria Chimica “Giulio Natta” Politecnico di Milano via Luigi Mancinelli 7 Milano 20133 Italy; ^4^ Dipartimento di Ingegneria Industriale Università degli Studi di Firenze via S. Marta 3 Firenze 50136 Italy; ^5^ Centro per la Condivisione della Strumentazione Scientifica dell'Università di Pisa – CISUP Lungarno Pacinotti 43/44 Pisa 56126 Italy

**Keywords:** adsorption, coordination polymers, NMR spectroscopy, nuclear relaxation, volatile organic compounds

## Abstract

1D coordination polymers (CPs) made of parallel, weakly interacting chains have been recently proposed for the selective adsorption of volatile organic compounds (VOCs), one of the main sources of air and water pollution. Since the efficiency of adsorption for VOC removal strongly depends on the specific and stable interactions between the volatile species and the CP framework, the characterization at the atomic level of host‐guest interactions and guest dynamics is of particular interest. Here, low‐ and high‐resolution solid‐state NMR experiments were applied to acquire information on ligand and guest dynamics for two 1D CPs (**1**‐ClBz and **2**‐ClBz), both containing a bispidine organic ligand, Hg(II) as a metal center, and chlorobenzene trapped within the framework, but featuring different topologies. By combining analyses of on‐resonance ^1^H free induction decays (FIDs), ^1^H and ^13^C longitudinal relaxation times, and ^2^H quadrupole echo spectra, it was found that chlorobenzene undergoes fast dynamics, mainly isotropic, in **1**‐ClBz, while it shows slower anisotropic motions in **2**‐ClBz, indicating stronger interactions between framework and adsorbate in the latter associated with the lower available free volume. Ligands are quite rigid for both CPs in the investigated frequency scale, apart from methyl groups, which undergo fast motions about their ternary symmetry axes.

## Graphical abstract

1D CPs are promising materials for the adsorption of VOCs. Solid‐state NMR was applied to characterize host‐guest interactions and dynamics of chlorobenzene in two CPs with different topologies. While ligand dynamics were similar in both CPs, chlorobenzene was found to undergo more restricted dynamics in the CP with polycatenated topology because of the smaller free volume and stronger interactions.

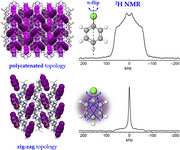

## 1 Introduction

Coordination polymers (CPs) are metal‐organic materials with long‐range ordered structure built by linking metal ions or clusters through coordination bonds with organic ligands.^[^ ^1^, ^2^ ^]^ Depending on whether the metal‐ligand connectivity extends along one, two, or three dimensions, CPs can be classified as 1D, 2D, and 3D, respectively.^[^ ^3^ ^]^ In the past decades, these materials have attracted considerable attention not only for their fascinating electronic, magnetic, and optical properties but also for their high porosity and the ability to finely tune their characteristics through ligand design, an advantage less readily achievable with traditional inorganic materials. In particular, CPs have been extensively explored for a wide range of applications, including gas adsorption and storage, catalysis, and sensing.^[^ ^4^, ^5^ ^]^ Metal‐organic frameworks (MOFs), that is, intrinsically porous CPs, usually with 2D or 3D dimensionalities, are considered to be the most promising materials thanks to their high crystallinity, multitude of different architectures, and large accessible free volume with controllable pore size.^[^ ^2^, ^6^, ^7^, ^8^, ^9^, ^10^ ^]^ Although previously regarded as less prominent compared to their higher‐dimensional counterparts, 1D CPs have recently gained recognition for their unique advantages, such as easier design, structural flexibility, and specific adsorption capabilities, which make them well‐suited for various practical applications.^[^ ^11^, ^12^, ^13^, ^14^, ^15^ ^]^ From a structural point of view, 1D CPs are composed of linear chains that, depending on the metal/ligand stoichiometry and the ligand shape and flexibility, can feature a multitude of different topologies, such as linear, zig‐zag, ladder, ribbon‐like, or helical.^[^ ^11^, ^16^ ^]^ Usually, these chains are packed in a three‐dimensional lattice via weak interchain interactions that impart a pronounced crystal flexibility and may affect the ability to respond to physical and/or chemical stimuli. This dynamic behavior is a defining feature of 1D CPs, often resulting in remarkable structural transformations upon guest adsorption.^[^ ^17^, ^18^, ^19^, ^20^, ^21^ ^]^

Recently, 1D CPs have been proposed for the selective adsorption of volatile organic compounds (VOCs).^[^ ^4^, ^22^ ^]^ VOCs are used as intermediates or solvents in many chemical processes and represent one of the major sources of air and water pollution. Hence, their removal is necessary to avoid their harmful effects on the environment and health. The efficiency of adsorption methods for VOC removal strongly depends on specific and stable interactions between the VOC and the host adsorbent framework. This renders the understanding at the atomic level of host‐guest interactions and the related guest dynamics vital for the elucidation of adsorption mechanisms and capabilities, in view of the design of optimized CPs for the selective adsorption of specific VOCs.

Solid‐state nuclear magnetic resonance spectroscopy (SSNMR) is one of the most powerful techniques for the investigation of structural and dynamic properties of solid adsorbent materials at the atomic scale, providing complementary structural information to long‐range characterization techniques, such as X‐ray diffraction (XRD), and unique dynamic information.^[^ ^23^, ^24^, ^25^, ^26^, ^27^ ^]^ Indeed, SSNMR has been successfully applied to investigate ligand structure and dynamics, guest dynamics, and host‐guest interactions in MOFs,^[^ ^28^, ^29^, ^30^, ^31^, ^32^, ^33^, ^34^, ^35^, ^36^, ^37^, ^38^, ^39^, ^40^, ^41^, ^42^, ^43^, ^44^, ^45^, ^46^, ^47^ ^]^ as well as interactions of VOCs with other solid sorbents.^[^ ^48^, ^49^ ^]^

**Scheme 1:**
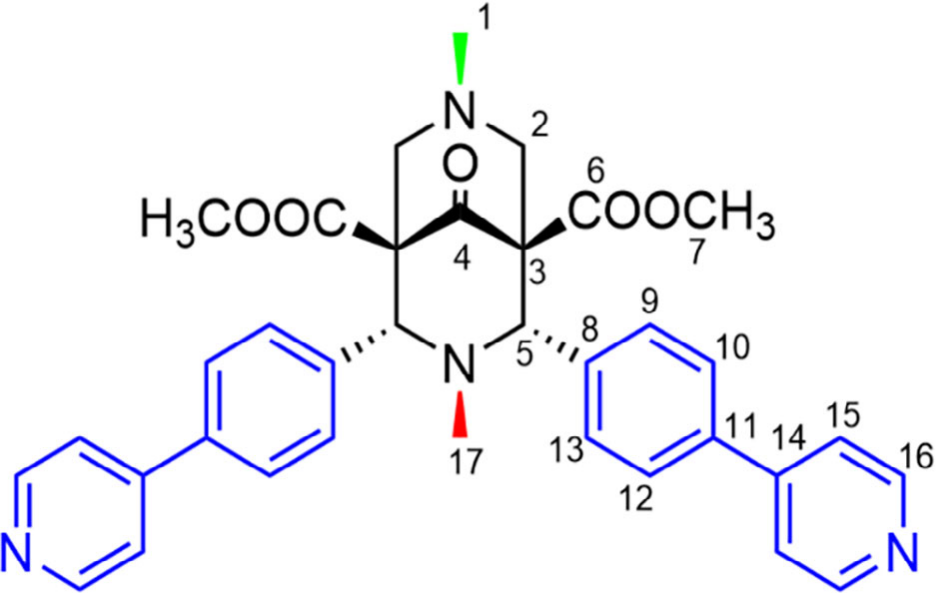
Chemical formula of the bispidine ligand **L** with numbering of carbon atoms.

**Figure 1:**
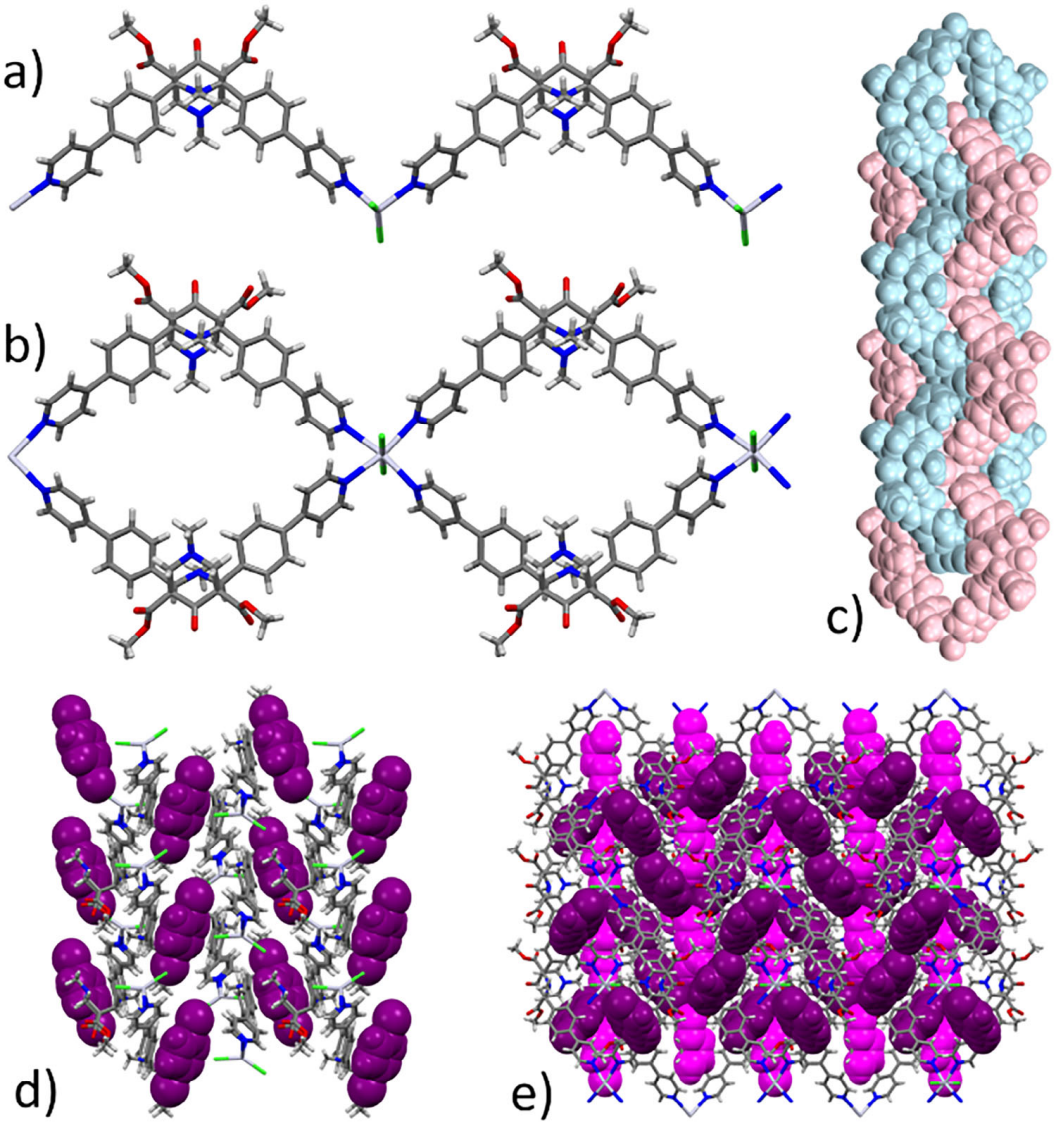
Views of a) zig‐zag array of **1**‐ClBz, b) ribbon‐like 1D chain of **2**‐ClBz and c) its polycatenated topology; packing view of d) **1**‐ClBz and e) **2**‐ClBz. Chlorobenzene molecules are reproduced in CPK styles and colored according to their crystallographic independence.

In the present work, SSNMR experiments were applied to two recently synthesized 1D CPs, **1**‐ClBz and **2**‐ClBz, constituted by the same ligand and metal ion components and guest solvent (i.e., chlorobenzene, considered as a VOC model), but differing in terms of stoichiometry and, more importantly, overall CP topology. Indeed, both CPs are made of the bispidine ligand **L** (Scheme 1), Hg(II) as the metal center, and chlorobenzene (ClBz) trapped within the framework. **1**‐ClBz is identified with the formula [**L**∙HgCl_2_]∙0.5ClBz and shows 1D linear arrays with zig‐zag topology (Figure 1a), whereas the formula of **2**‐ClBz is [**L**∙Hg_0.5_Cl]∙1.5ClBz, and it is characterized by ribbon‐like polycatenated arrays (Figure 1b,c).^[^ ^50^ ^]^ In both CPs, **L** functions as a bis‐monodentate ligand through its pyridinic sidearms (Figure 1a,b), whereas the central aliphatic nitrogen atoms are not involved in metal coordination.

In the case of zig‐zag topology, Hg(II) ions are tetracoordinated in a distorted trigonal pyramidal geometry, whereas in the case of polycatenated topology, Hg(II) ions are hexacoordinated in an octahedral geometry. In both topologies, chlorobenzene molecules occupy the void interchain spaces of the CPs. According to XRD results at 100 K, in **1**‐ClBz chlorobenzene molecules are sandwiched between two side arms of different arrays with two different orientations (related by an inversion center). Their orientation is nearly parallel to the mean plane defined by the 4‐phenyilpyridine sidearm moieties of the ligand and fill noninterconnected pockets (Figure 1d). Instead, in **2**‐ClBz, chlorobenzene molecules are arranged both in a head‐to‐tail and in a T‐stacked fashion in interchain channels (Figure 1e). Chlorobenzene molecules are characterized by orientational (in **1**‐ClBz and **2**‐ClBz) and positional (in **2**‐ClBz) disorder.^[^ ^50^ ^]^ In light of these structural features, the investigated CPs offer the opportunity to unravel the role of framework topology and metal coordination environment, the two key factors differentiating **1**‐ClBz and **2**‐ClBz, in modulating host and guest dynamics and host‐guest interactions.

Here, ^1^H and ^13^C SSNMR spectra were recorded under magic angle spinning (MAS) conditions to investigate the structural and dynamic properties of the ligands in the two CPs. Analyses of ^1^H spin‐spin and spin‐lattice relaxation times^[^ ^51^, ^52^ ^]^ as a function of temperature allowed dynamic processes of both ligands and chlorobenzene to be probed, while the dynamics of the ligands was more specifically investigated by analyzing ^13^C spin‐lattice relaxation times.^[^ ^53^ ^]^ In fact, internal and molecular motions may affect nuclear relaxation at peculiar frequencies by modulating nuclear interactions. While ^1^H transverse magnetization decay is mainly influenced by motions with frequencies in the 10^4^–10^5^ s^−1^ range modulating the homonuclear dipolar interaction, ^1^H and ^13^C longitudinal relaxation is sensitive to dynamic processes with rates close to the relevant Larmor frequency (on the order of 10^8^–10^9^ s^−1^).^[^ ^27^ ^]^ Line shape analysis of ^2^H static NMR spectra recorded at different temperatures was instead applied to selectively investigate the guest dynamics exploiting chlorobenzene‐d_5_. In fact, ^2^H SSNMR represents a reliable method for probing the dynamics of guests in confined geometries, including microporous and mesoporous materials such as zeolites or MOFs, providing information on the rate and geometry of local reorientational motions and jumps among adsorption sites.^[^ ^31^, ^33^, ^35^, ^38^, ^39^, ^40^, ^44^, ^54^ ^]^ In the case of **1**‐ClBz and **2**‐ClBz, the availability of well‐characterized crystallographic structures allows the correlation between the dynamic properties determined by SSNMR and structural features, thus offering a deep and reliable way to explore how subtle framework differences affect VOC mobility and interaction strength in 1D CPs, with implications for the rational design of selective adsorbent materials.

## 2 Experimental

### 2.1 Materials

Microcrystalline powders of **1**‐ClBz and **2**‐ClBz were prepared by following a published procedure.^[^ ^50^ ^]^ Samples with deuterated chlorobenzene (**1**‐ClBz_d_5_ and **2**‐ClBz_d_5_) were prepared analogously by replacing chlorobenzene with chlorobenzene‐d_5_ (Merck, 99 atom % D). P‐XRD and ^1^H‐NMR characterization is provided in the Supporting Information (Section S1).

### 2.2 Methods

^1^H and ^13^C high‐resolution SSNMR experiments were carried out on a Bruker Avance Neo 500 spectrometer, operating at the Larmor frequencies of 500.13 and 125.76 MHz for ^1^H and ^13^C nuclei, respectively, equipped with a 4 mm Cross‐Polarization/Magic Angle Spinning (CP/MAS) probe. ^1^H and ^13^C 90° pulse durations were 4.25 and 3.85 µs, respectively. ^1^H Direct Excitation (DE)/MAS spectra were recorded, accumulating 16 scans with a recycle delay of 6 s for **1**‐ClBz and **1**‐ClBz_d_5_ and of 12 s for **2**‐ClBz and **2**‐ClBz_d_5_. ^1^H‐^13^C CP experiments were recorded using contact time values ranging from 40 µs to 8 ms, acquiring 1200 scans with a recycle delay of 4 s for **1**‐ClBz and **1**‐ClBz_d_5_ and of 6 s for **2**‐ClBz and **2**‐ClBz_d_5_. ^13^C longitudinal relaxation times (*T*_1_) were measured on **1**‐ClBz between 30 and 72 °C using the Torchia pulse sequence^[^ ^53^ ^]^ exploiting ^1^H‐^13^C CP with a contact time of 4 ms, with a recycle delay of 4 s and variable relaxation delay values ranging from 10 ms to 40 s, and accumulating 360 scans. In all experiments, a MAS spinning frequency of 15 kHz was employed. Dry air was used as spinning and heating/cooling gas. Temperature was calibrated using the ^207^Pb chemical shift of Pb(NO_3_)_2_ and controlled with ± 0.1 °C precision. Spectra were processed using Bruker TopSpin and MestReNova software. Spectra acquired for ^13^C *T*_1_ measurements were deconvoluted using the SPORT‐NMR software.^[^ ^55^ ^]^

Time‐domain SSNMR experiments were carried out using a STELAR PC‐NMR spectrometer, operating at the ^1^H Larmor frequency of 20.8 MHz. The 90° pulse duration was 3.3 µs. On resonance free induction decays (FIDs) were recorded using the magic sandwich echo (MSE) pulse sequence.^[^ ^51^, ^52^ ^]^ The total echo duration was set to τ_MSE_ = 6(2τ_90°_ + 4τ_φ_),^[^ ^52^ ^]^ with τ_φ_ = 4.4 µs and τ_90°_ = 3.3 µs. Recycle delay values between 1 and 3.5 s were used, and 64 scans were accumulated. *T*_2_ measurements were also carried out using the Carr‐Purcell‐Meiboom‐Gill (CPMG) pulse sequence^[^ ^56^, ^57^ ^]^ acquiring 2000 echoes with an echo delay of 50 µs; 512 scans were accumulated with a recycle delay of 4 s. ^1^H *T*_1_ relaxation time measurements were performed using the inversion recovery pulse sequence coupled with solid echo. Experiments were performed between −100 and 20 °C using nitrogen as heating/cooling gas; temperature was controlled with ± 0.1 °C precision. Recycle delay values of 1 to 3.5 s were used, and 16 scans were accumulated. ^1^H FIDs and longitudinal relaxation data were analyzed using purposely written programs in Mathematica.^[^ ^58^ ^]^

^2^H static spectra were recorded on **1**‐ClBz_d_5_ and **2**‐ClBz_d_5_ using a Varian InfinityPlus 400 spectrometer equipped with an H‐F/X goniometric probe, operating at the ^2^H Larmor frequency of 61.35 MHz. Spectra were acquired using the quadrupole echo pulse scheme^[^ ^59^ ^]^ with an echo delay of 30 µs. The ^2^H 90° pulse duration was 3 µs. A recycle delay of 2 s was used, and 1800 scans were accumulated for **1**‐ClBz_d_5_, while 400 transients were accumulated for **2**‐ClBz_d_5_ using a recycle delay of 8 s. Experiments were performed from −70 °C to 20 °C using nitrogen as cooling gas. Temperature was controlled with ± 0.1 °C precision. ^2^H spectra were simulated using the NMR Weblab software^[^ ^60^ ^]^ combined with a code written in Mathematica.^[^ ^58^ ^]^

## 3 Results and Discussion

### 3.1 ^1^H and ^13^C High‐Resolution SSNMR Spectra

^1^H and ^13^C SSNMR spectra were recorded on **1**‐ClBz and **2**‐ClBz and the corresponding samples with perdeuterated chlorobenzene (**1**‐ClBz_d_5_ and **2**‐ClBz_d_5_) to investigate the ligands’ structure and acquire first qualitative information on ligand and adsorbate dynamics.

**Figure 2:**
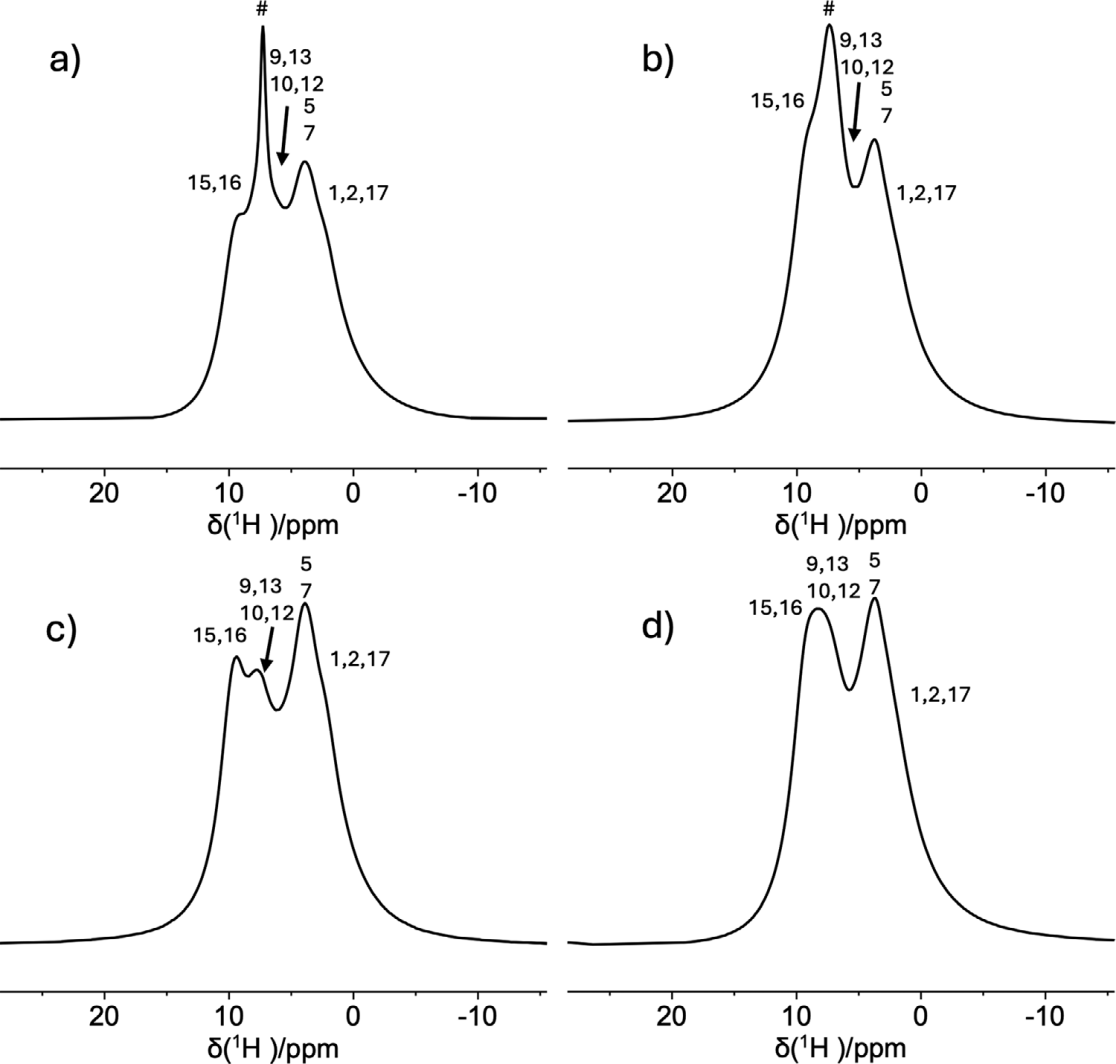
^1^H DE/MAS spectra of **1**‐ClBz a), **2**‐ClBz b), **1**‐ClBz_d_5_ c), and **2**‐ClBz_d_5_ d); the assignment numbering refers to the ligand in Scheme 1. # indicates the signal assigned to chlorobenzene protons. The spectra were recorded at 25 °C using a spinning frequency of 15 kHz.

In the ^1^H DE/MAS spectra of the investigated samples, shown in Figure 2, signals from aromatic and aliphatic moieties of the ligands are observed; in the spectra of **1**‐ClBz and **2**‐ClBz, an additional signal ascribable to the protons of chlorobenzene is found at about 7.2 ppm. The latter signal is much narrower in the spectrum of **1**‐ClBz, indicating a higher mobility of chlorobenzene in the CP with zig‐zag topology with respect to that with polycatenated topology. Indeed, the main cause of line broadening in ^1^H spectra is the residual homonuclear dipolar interaction that is stronger in rigid fractions, whereas it is partially or totally averaged out when fast anisotropic or isotropic motions, respectively, occur.

**Figure 3:**
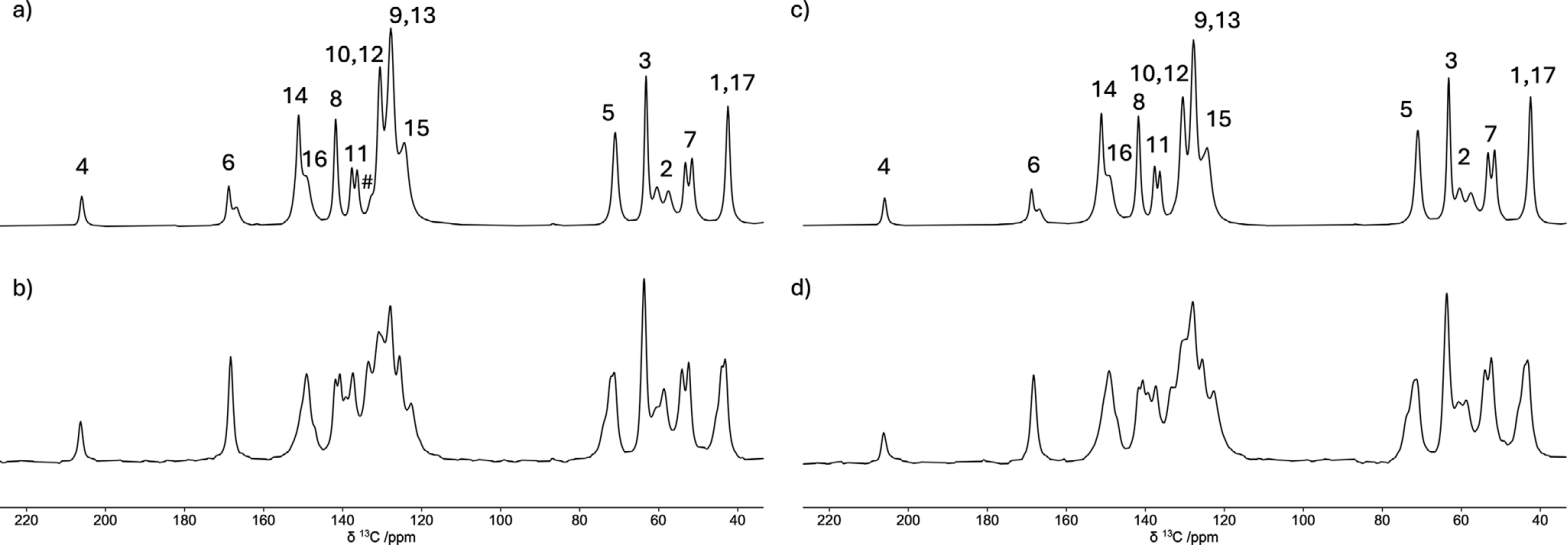
^13^C CP/MAS spectra of **1**‐ClBz a), **2**‐ClBz b), **1**‐ClBz_d_5_ c), and **2**‐ClBz_d_5_ d) recorded with a contact time of 2 ms; the assignment numbering refers to Scheme 1. # indicates the signal assigned to chlorobenzene carbons. The spectra were recorded at 25 °C using a spinning frequency of 15 kHz.

^13^C CP/MAS spectra were also recorded on all the investigated samples with contact time (ct) values ranging from 40 µs to 8 ms (Figures S4‐S5); the spectra recorded with ct = 2 ms are shown and assigned in Figure 3. As it can be observed, spectra of the same polymer containing either chlorobenzene or chlorobenzene‐d_5_ slightly differ, and signals from chlorobenzene carbons are not clearly observed, except for the small signal at 132.9 ppm in the spectrum of **1**‐ClBz. This finding is not surprising considering the low chlorobenzene content and the overlap of peaks from chlorobenzene carbons and ligands’ aromatic carbons.

**2**‐ClBz and **2**‐ClBz_d_5_ show more complex spectra with respect to **1**‐ClBz and **1**‐ClBz_d_5_, with multiple signals for some aliphatic and aromatic carbons associated with different local environments. In fact, the ligand exhibits a different arrangement in the different topologies characterizing the two CPs, where it clearly experiences different chemical environments. In particular, in **2**‐ClBz the ligand is partially inserted into the macrocyclic cavity of the ribbon‐like arrays of the polycatenated structure. On the other hand, based on analysis of distances and angles provided by SC‐XRD data,^[^ ^50^ ^]^ there is no significant difference between **1**‐ClBz and **2**‐ClBz in terms of interchain interactions, which can be considered weak in both CPs.

### 3.2 ^1^H FID Analysis and $T_{2}^{*}$ Measurements

**Figure 4:**
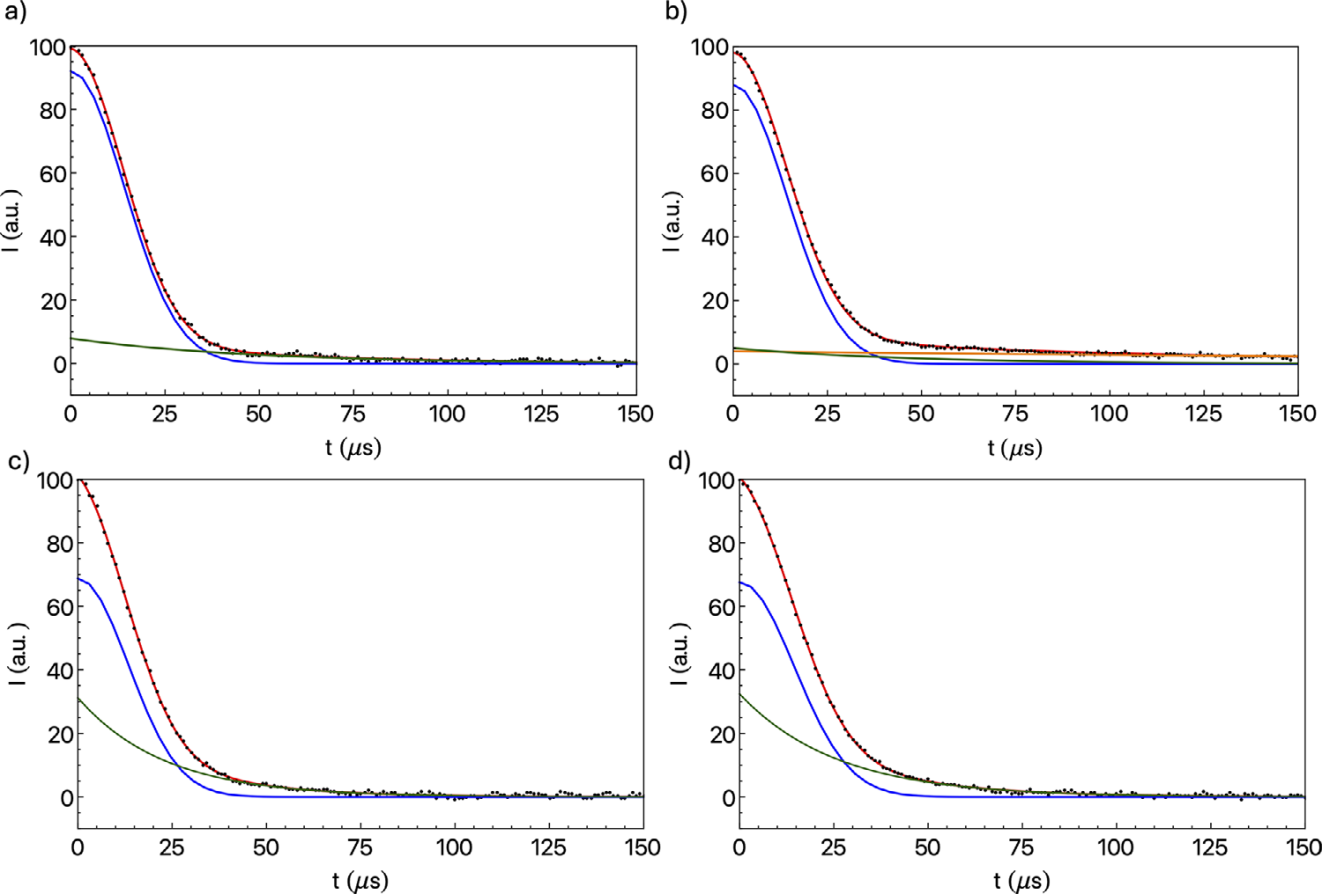
Experimental (black points) and calculated (red lines) ^1^H FIDs of a) **1**‐ClBz_d_5_, b) **1**‐ClBz, c) **2**‐ClBz_d_5_, and d) **2**‐ClBz at 20 °C. Single functions used to reproduce the FIDs according to equations (1), (2) are given as blue (Gaussian), green (exponential 1), and orange (exponential 2) lines.

On‐resonance ^1^H FIDs were recorded and analyzed for all samples at different temperatures between −100 and 20 °C to investigate the mobility of chlorobenzene and polymer ligands; examples are shown in Figure 4. In fact, the decay in time of the ^1^H NMR signal can give information on molecular mobility, being mainly governed by ^1^H‐^1^H dipolar couplings fluctuating because of internal and molecular motions. More mobile molecules or molecular moieties show a more slowly decaying signal, and different decaying functions can be expected depending on the motion geometry and dipolar coupling network.^[^ ^51^ ^]^

For all the investigated samples, a fast‐decaying component, associated with protons in moieties with restricted mobility, dominates the FIDs, accompanied by a smaller slow‐decaying component arising from protons in more mobile environments. In order to better identify and quantify proton fractions with different mobility, FIDs were analyzed using a discrete approach, that is, by reproducing the experimental decays with a linear combination of analytical functions characterized by different effective spin‐spin relaxation times ($T_{2i}^{*}$).^[^ ^61^, ^62^ ^]^ The minimum number of functions that satisfactorily reproduces the signal decays was employed. In particular, a Gaussian function with a spin‐spin relaxation time $T_{2g}^{*}$ was used in all cases to represent the fast decay, while an exponential function was employed for the slower decay with relaxation time $T_{2e}^{*}$ for all samples but **1**‐ClBz, for which two exponential functions (with effective spin‐spin relaxation times $T_{2e1}^{*}$ and $T_{2e2}^{*}$,) were necessary to account for the slow decay. Therefore, Equation 1 was used as a fitting function for **2**‐ClBz, **1**‐ClBz_d_5_, and **2**‐ClBz_d_5_, and Equation 2 for **1**‐ClBz:

(1) $$I\left(t\right)=\frac{I\left(0\right)}{100}\left[w_{g}\cdot e^{-{\left(\frac{t}{T_{2g}^{*}}\right)}^{2}}+w_{e}\cdot e^{-\left(\frac{t}{T_{2e}^{*}}\right)}\right]$$

(2) $$I\left(t\right)=\frac{I\left(0\right)}{100}\left[w_{g}\cdot e^{-{\left(\frac{t}{T_{2g}^{*}}\right)}^{2}}+w_{e1}\cdot e^{-\left(\frac{t}{T_{2e1}^{*}}\right)}+w_{e2}\cdot e^{-\left(\frac{t}{T_{2e2}^{*}}\right)}\right]$$

where *w*_i_ is the weight percentage of the i‐th function (with i = g, e, e1, or e2). With these functions, a good reproduction of all FIDs was obtained; examples are shown in Figure 4, while best‐fit parameters are reported in Tables 1, 2.

**Table 1:** Values of $T_{2}^{*}$ and weights of the Gaussian and exponential components (see Equations (1), (2)) obtained from the analysis of the on‐resonance ^1^H FIDs at the indicated temperatures for **1**‐ClBz_d_5_ and **1**‐ClBz. In both cases, $T_{2g}^{*}$ was fixed at 20 µs at all temperatures. For **1**‐ClBz, $T_{2e2}^{*}$ was fixed at 300 µs at all temperatures and $T_{2e1}^{*}$ was considered equal to the $T_{2e}^{*}$ value obtained for **1**‐ClBz_d_5_ at each temperature.

	1‐ClBz_d_5_			1‐ClBz		
T [°C]	$T_{2e}^{*}$ [µs]	*w*_g_ [%]	*w*_e_ [%]	*w*_g_ [%]	*w*_e1_ [%]	*w*_e2_ [%]
−100	24	90	10	77	22	1
−90	25	87	13	83	15	2
−80	27	87	13	88	10	2
−70	27	87	13	86	11	3
−60	28	86	14	89	7	4
−50	31	87	13	87	9	4
−40	36	90	10	92	4	4
−30	35	90	10	90	7	3
−20	42	92	8	91	6	3
−10	38	91	9	89	8	3
0	43	91	9	90	7	3
10	42	90	10	89	7	4
20	47	91	9	86	11	3

**Table 2:** Values of $T_{2}^{*}$ and weights of the Gaussian and exponential components (see Equation 1) obtained from the analysis of the on‐resonance ^1^H FIDs at the indicated temperatures for **2**‐ClBz_d_5_ and **2**‐ClBz. In both cases, $T_{2g}^{*}$ was fixed at 19 µs at all temperatures.

	2‐ClBz_d_5_			2‐ClBz		
T [°C]	$T_{2e}^{*}$ [µs]	*w*_g_ [%]	*w*_e_ [%]	$T_{2e}^{*}$ [µs]	*w*_g_ [%]	*w*_e_ [%]
−100	21	70	30	20	73	27
−90	21	73	27	22	78	22
−80	20	71	29	25	83	17
−70	21	74	26	26	84	16
−60	21	75	25	29	86	14
−50	20	76	24	28	85	15
−40	21	75	25	27	83	17
−30	21	75	25	26	83	17
−20	24	79	21	26	81	19
−10	22	74	26	27	80	20
0	22	74	26	28	78	22
10	24	73	27	28	74	26
20	23	68	32	26	67	33

For **1**‐ClBz_d_5_ and **2**‐ClBz_d_5_, the ^1^H FID analysis gives insight into the dynamics of the sole ligand, given the absence of protons in the adsorbate. Two fractions of ligand protons with slightly different mobility were found: one main fraction associated with the Gaussian function and a smaller one reproduced by an exponential function. Since the $T_{2}^{*}$ values associated with the two components are of the same order of magnitude (a few tens of µs), correlation was found among parameters in the fitting, especially at the lower temperatures. Therefore, the fitting was performed by fixing $T_{2g}^{*}$ at the same value at all temperatures. In the case of **1**‐ClBz_d_5_, $T_{2g}^{*}$ was fixed at 20 µs; with this assumption, $T_{2e}^{*}$ values increasing with temperature from 24 to 47 µs were found for the exponential component. The relative weights of the Gaussian and exponential components were *w*_g_:*w*_e_ ≅ 90:10 at all the investigated temperatures (Table 1). Overall, the FID analysis indicates that ligands of **1**‐ClBz_d_5_ undergo restricted dynamics in the investigated temperature range, with only a small fraction being progressively mobilized above −60 °C. In the case of **2**‐ClBz_d_5_, $T_{2g}^{*}$ was fixed at 19 µs; consequently, the *w*_g_:*w*_e_ ratio was found to be about 75:25 at most temperatures, with $T_{2e}^{*}$ slightly increasing from 21 to 24 µs by increasing the temperature (Table 2). These findings indicate that **2**‐ClBz_d_5_ ligands undergo quite restricted dynamics at all the investigated temperatures, more restricted than that of **1**‐ClBz_d_5_ ligands.

For **1**‐ClBz and **2**‐ClBz, chlorobenzene protons also contribute to the ^1^H FIDs. By comparing the FIDs of **1**‐ClBz and **1**‐ClBz_d_5_ (see, for instance, Figure 4a,b), it is obvious that a longer component is present for the sample with chlorobenzene. Therefore, assuming that the polymer ligands behave in the same way in both samples, FIDs of **1**‐ClBz were reproduced using Equation 2 by fixing $T_{2g}^{*}$ and $T_{2e1}^{*}$ at the values obtained for **1**‐ClBz_d_5_, while the weights of all decay functions and $T_{2e2}^{*}$ were left as free fitting parameters. The additional slow‐decaying exponential component, ascribed to chlorobenzene protons, is characterized by a time constant, $T_{2e2}^{*}$, on the order of 300 µs and a weight (*w*_e2_) of 2–4% (Table 1), slightly lower than expected on the basis of the sample composition (∼7%).

Since long‐decaying signals can be affected by magnetic field inhomogeneity in MSE measurements, CPMG experiments^[^ ^56^, ^57^ ^]^ were performed to more accurately determine the spin‐spin relaxation time associated with chlorobenzene protons. Measurements performed between −40 and 20 °C gave magnetization decays reproducible with a sum of two exponential functions (an example is shown in Figure S6), one with a longer $T_{2}^{*}$ of 40–50 ms and the other with a shorter $T_{2}^{*}$ of 1–2 ms, which can both be associated with fast, mainly isotropic dynamics of chlorobenzene. Since the whole CPMG signal reasonably arises from the slow‐decaying exponential component (e2) of the FID, the two exponential functions are associated with 1 and 3% of total protons, respectively.

In the case of **2**‐ClBz, a Gaussian and an exponential functions (Equation 1) were sufficient to reproduce the FIDs well (Figure 4d), with values of effective relaxation times and weights reported in Table 2. By comparing these parameters with those obtained for **2**‐ClBz_d_5_ (Table 2), larger *w*_g_ values are found for **2**‐ClBz, indicating that the solvent protons contribute to the Gaussian fraction. Therefore, FID analyses clearly indicate quite restricted dynamics of chlorobenzene in the CP with polycatenated topology, at variance with fast isotropic dynamics, at least for a part of chlorobenzene molecules, in that with zig‐zag topology.

### 3.3 Ligands’ Dynamics by ^1^H and ^13^C *T*_1_ Analysis

The ligand dynamics were investigated in more detail by measuring and analyzing ^1^H *T*_1_’s as a function of temperature for all samples and ^13^C *T*_1_’s for **1**‐ClBz.

**Figure 5:**
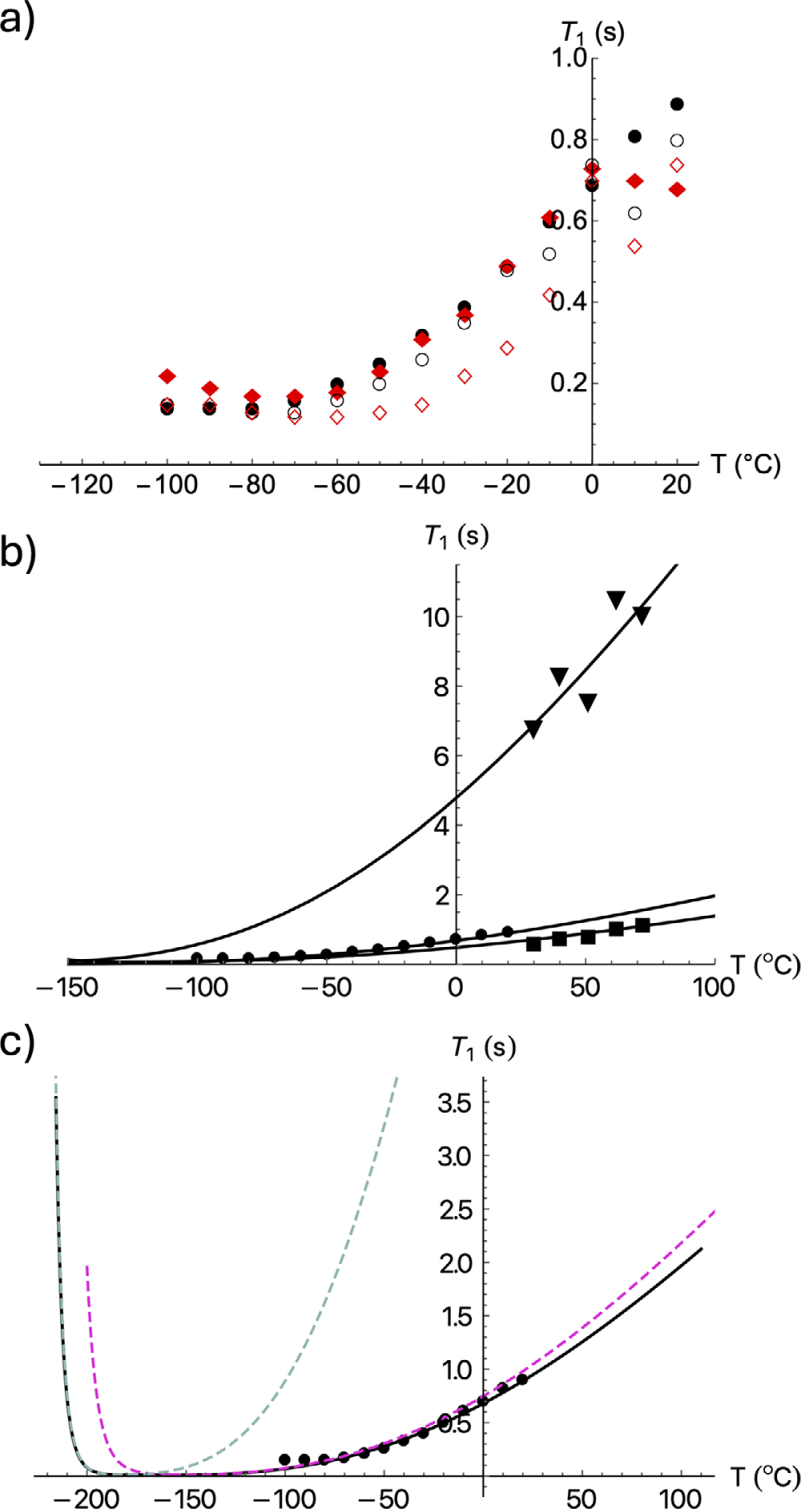
a) Experimental ^1^H *T*_1_ values of **1**‐ClBz (black full circles), **1**‐ClBz_d_5_ (black empty circles), **2**‐ClBz (red full diamonds), and **2**‐ClBz_d_5_ (red empty diamonds) versus temperature. b) Experimental (symbols) and calculated (black lines) ^13^C *T*_1_ values of C1/C17 (black full squares) and C7 (black full triangles) and ^1^H *T*_1_ values (black full circles) of **1**‐ClBz versus temperature. c) Experimental (symbols) and calculated (black line) ^1^H *T*_1_ values of **1**‐ClBz versus temperature; the calculated contributions to ^1^H spin‐lattice relaxation due to the motions of NCH_3_ (dashed fuchsia line) and COOCH_3_ (dashed light green line) methyl groups are also shown.

^1^H *T*_1_’s were measured between −100 and 20 °C at the Larmor frequency of 20.8 MHz. At all temperatures, a single *T*_1_ value was determined for each sample, indicating that spin diffusion has completely averaged the longitudinal relaxation times of all protons in the sample. Short *T*_1_ values that increase with temperature were determined for all samples (Figure 5a and Table S1), indicating that moieties undergoing activated motions with frequencies slightly higher than the ^1^H Larmor frequency are present. In fact, even if the measured *T*_1_ values are determined in principle by all motions in the sample, in practice only motions with a characteristic frequency on the order of the Larmor one give a nonnegligible contribution to relaxation (see theoretical expressions of *T*_1_ reported below). In the investigated cases, at all temperatures the ratio between the ^1^H *T*_1_ values measured for **1**‐ClBz_d_5_ and **1**‐ClBz (and, analogously, for **2**‐ClBz_d_5_ and **2**‐ClBz) is similar and close to the ratio between the number of protons in the corresponding samples (see Section S4 of Supporting Information). This supports the hypothesis that a single motion for a ligand moiety drives longitudinal relaxation of all protons in the system.

To single out the motion(s) responsible for relaxation in the MHz regime, ^13^C *T*_1_’s were measured on **1**‐ClBz at the Larmor frequency of 125 MHz, varying the temperature from 30 to 72 °C, using the Torchia pulse sequence^[^ ^53^ ^]^ and fitting the integral intensities of all carbon signals determined using a deconvolution procedure (see Supporting Information, Section S4); the obtained values are reported in Table S2, Figure 5b. Differently from protons, every chemically distinct ^13^C nucleus exhibits its own relaxation time, determined by the motion(s) of the ligand fragment to which it belongs. In particular, *T*_1_’s affected by motions with frequencies of the order of the ^13^C Larmor frequency will present a minimum in their trend versus temperature, while *T*_1_ values increase or decrease with increasing the temperature when the motion frequency is respectively higher or lower than the Larmor one. Therefore, some preliminary conclusions about the dynamics of ligand fragments in the MHz regime can be drawn by looking at the *T*_1_ values and their trends with temperature. For all carbons, except for the methyl ones, very long relaxation times (>30 s) were found. On the other hand, *T*_1_ values on the order of 1 s and 10 s, increasing with temperature, were determined for C1/C17 and C7 carbons, respectively (Table S2, Figure 5b). Considering that the relaxation of methyl ^13^C nuclei is principally driven by the modulation of the dipolar interactions with ^1^H nuclei (vide infra), these values are compatible with the reorientation of the methyl groups around their threefold symmetry axis.^[^ ^63^, ^64^, ^65^ ^]^ In particular, the characteristic frequency of the reorientation is closer to the ^13^C Larmor frequency for the NCH_3_ than for the COOCH_3_ methyl group, although higher than the Larmor frequency in both cases. On the other hand, all the other moieties of the ligands do not experience any motion in the MHz regime.

Considering what was found for ^13^C *T*_1_’s, the values and trends with temperature of ^1^H *T*_1_’s of **1**‐ClBz can tentatively be ascribed to reorientational motions of methyl groups in NCH_3_ and COOCH_3_ moieties. To corroborate this hypothesis and obtain quantitative information on these motions, trends with temperatures of ^1^H *T*_1_’s and ^13^C *T*_1_’s of C1/C17 and C7 of **1**‐ClBz were globally analyzed in terms of equations derived from the relaxation theory.^[^ ^27^ ^]^ By considering as main relaxation mechanism the modulation of the heteronuclear ^1^H‐^13^C interaction between directly bonded nuclei, the longitudinal relaxation rate of each carbon can be written as:

(3) $$\;\frac{1}{T_{1C}\left(i\right)}=\frac{N}{20}\;C_{\mathrm{HC}}^{2}\left(i\right)\left[J\left(\omega_{H}-\omega_{C}\right)+3J\left(\omega_{C}\right)+4J\left(\omega_{H}+\omega_{C}\right)\right]$$

where *ω*_Η_ and *ω*_C_ are the ^1^H and ^13^C Larmor frequencies, respectively, *N* is the number of protons directly bonded to carbon *i*, and *C*_HC_(*i*) is the heteronuclear dipolar coupling constant given by:

(4) $$C_{\mathrm{HC}}\left(i\right)=\frac{\mu_{0}}{4\pi }\;\gamma_{H}\gamma_{C}\hbar \frac{1}{r_{\mathrm{CH}}^{3}\left(i\right)}$$

with *r*_CH_(*i*) being the ^1^H‐^13^C internuclear distance for carbon *i*.

For ^1^H *T*_1_’s, instead, longitudinal relaxation is mainly determined by the modulation of homonuclear ^1^H‐^1^H dipolar interactions. Also taking into account the effect of spin diffusion, Equation 5 can be used to express the average relaxation rate:

(5) $$\;\langle \frac{1}{T_{1H}}\rangle =K_{H}\;\sum_{i}f^{i}\left[J^{i}\left(\omega_{H}\right)+4J^{i}\left(2\omega_{H}\right)\right]$$

where *K*_H_ is a constant related to the strength of the homonuclear dipolar coupling and $f^{i}$ is the fractional weight of protons contributing to the *i*‐th motion. The spectral densities, J(ω), are expressed using Lorentzian functions of the type:

(6) $$J\left(\omega \right)=\frac{2\tau_{c}}{1+\omega^{2}\tau_{c}^{2}}\;$$

corresponding to an exponential correlation function of the motion with a correlation time *τ*_c_. Finally, for each motion considered in the analysis, the dependence of the correlation time on temperature is expressed using the Arrhenius equation:

(7) $$\;\tau_{c}=\tau_{\infty }\;e^{\frac{E_{a}}{kT}}$$

where $\tau_{\infty }$ is the correlation time at infinite temperature and *E*_a_ is the activation energy of the motion.

Equations (3), (4) were used for the analysis of the C1/C17 and C7 ^13^C *T*_1_’s, considering the reorientation of the NCH_3_ and COOCH_3_ methyl groups around their C_3_ symmetry axes, with *N* = 3 and *r*_CH_ **= 1.09 Å. Both motions were also considered to govern the spin‐lattice relaxation of ^1^H nuclei; the analysis of ^1^H *T*_1_’s was therefore carried out using Equation 8:

(8) $$\begin{array}{ccc} \;\langle \frac{1}{T_{1H}}\rangle & = & K_{H}\;\{f^{NCH3}\cdot [J^{NCH3}\left(\omega_{H}\right)+4J^{NCH3}\left(2\omega_{H}\right)]\\ & & +\;f^{COOCH3}\cdot \left[J^{COOCH3}\left(\omega_{H}\right)+4J^{COOCH3}\left(2\omega_{H}\right)\right]\} \end{array}$$

with *K*_H_ as a fit parameter, and $f^{NCH3}$ and $f^{COOCH3}$ fixed equal to 0.16 on the basis of the **1**‐ClBz formula (see Section S4). The *T*_1_ values of the remaining protons in the ligand and in chlorobenzene were assumed infinitely long. A global fitting of ^1^H *T*_1_ and ^13^C *T*_1_ values of C1/C17 and C7 was performed with Lorentzian spectral densities (Eq. 6) and correlation times expressed as a function of temperature as in Equation 7, with activation energy and correlation time at infinite temperature of each motion as fitting parameters. A good reproduction of the experimental data was obtained (Figure 5b) with the optimized parameters shown in Table S3. As it can be observed from the individual contributions to ^1^H relaxation coming from the reorientations of NCH_3_ and COOCH_3_ methyl groups (Figure 5c), the reorientation of the NCH_3_ methyl groups constitutes the relaxation sink for all protons at the experimental temperatures and Larmor frequency here considered. Activation energy values of 8.4 and 9.1 kJ/mol were obtained for the motions of the methyl groups of COOCH_3_ and NCH_3_, respectively. These values are on the order of those found for methyl groups by other authors.^[^ ^63^, ^64^ ^]^ Correlation times at room temperature (2.4 and 23 ps) indicate faster rotation for the COOCH_3_ than for the NCH_3_ methyl, as previously qualitatively inferred from the ^13^C *T*_1_ values.

By considering the similar ^1^H *T*_1_ trends observed for **2**‐ClBz and **1**‐ClBz and the ratio between ^1^H *T*_1_ values of **2**‐ClBz_d_5_ and **2**‐ClBz (see discussion in Section S4), we could infer that the motion of the NCH_3_ methyl groups is the relaxation sink also for protons of **2**‐ClBz at the investigated Larmor frequency and temperatures.

### 3.4 Dynamics of Adsorbed Chlorobenzene by Line Shape Analysis of Static ^2^H NMR Spectra

**Figure 6:**
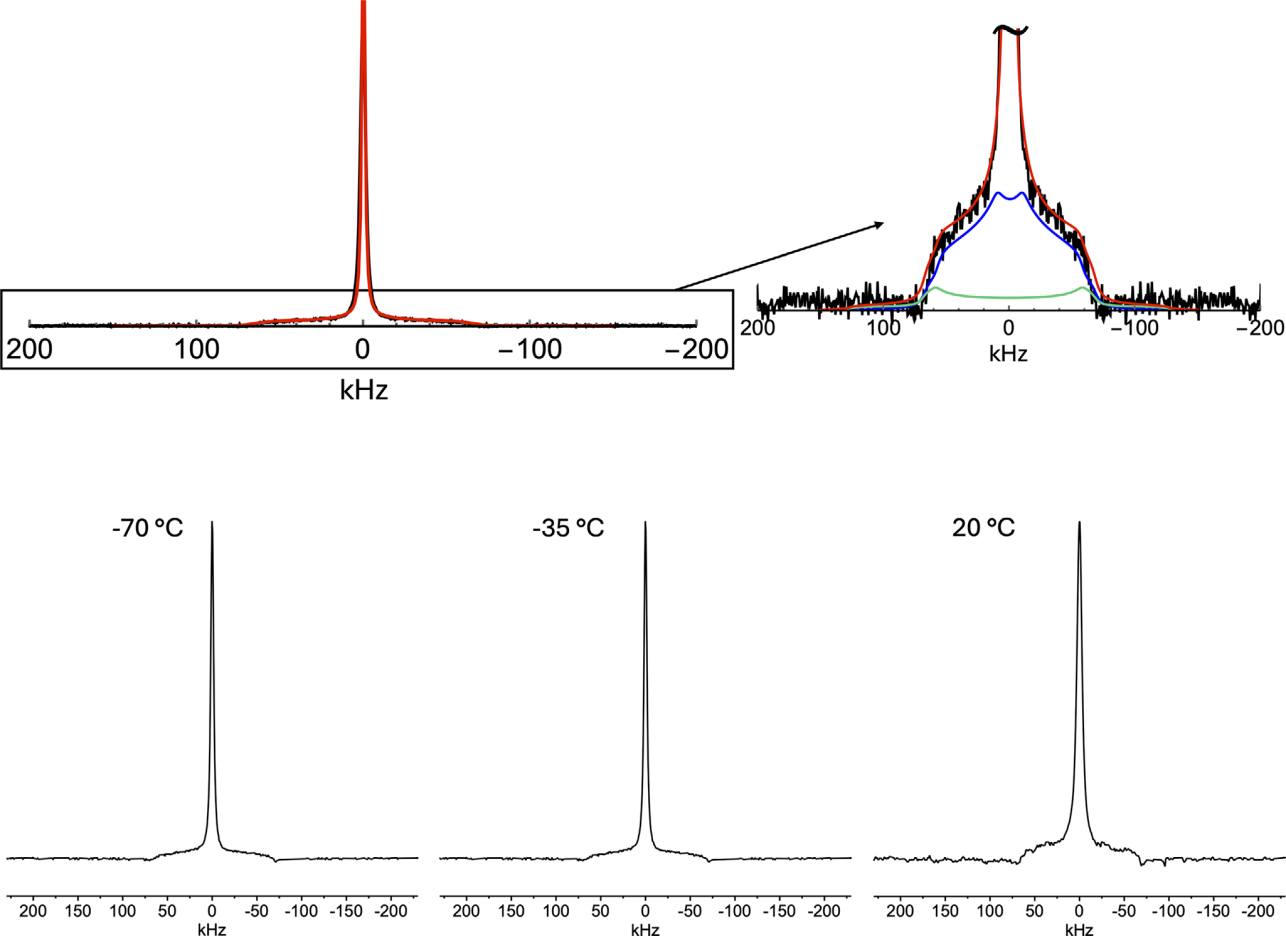
Bottom: ^2^H quadrupole echo NMR spectra of **1**‐ClBz_d_5_ at the indicated temperatures. Top: Experimental (black line) and simulated (red line) ^2^H quadrupole echo NMR spectrum of **1**‐ClBz_d_5_ at −70°C. The sub‐spectra associated with fast π‐flip and Pake patterns are shown in blue and green, respectively.

**Figure 7:**
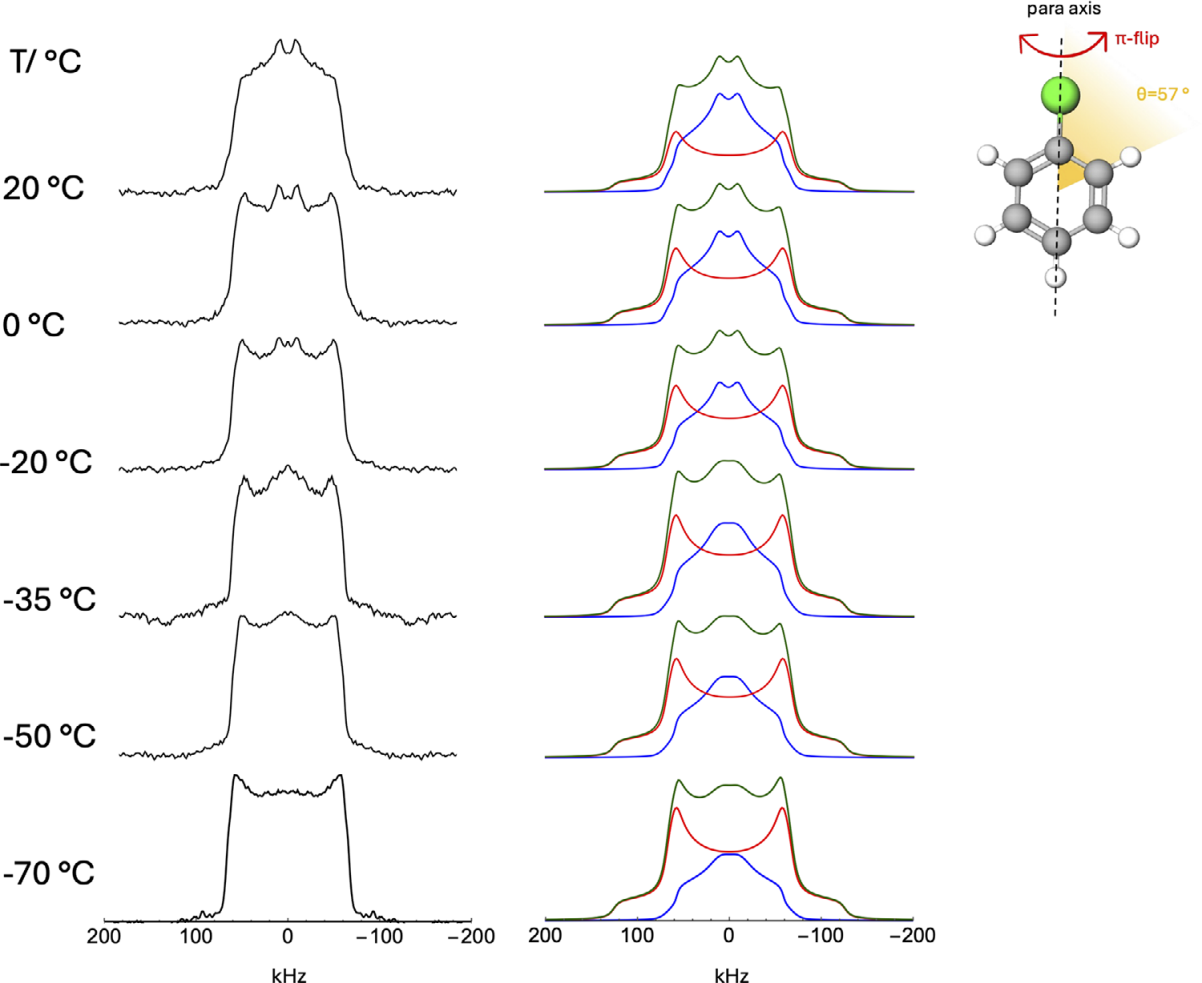
Left: experimental ^2^H quadrupolar echo spectra of **2**‐ClBz_d_5_ at the indicated temperatures; right: corresponding simulated spectra (green lines). The sub‐spectra associated with fast π‐flip and Pake patterns are shown in blue and red, respectively. Inset: sketch of the π‐flip for the chlorobenzene molecule about the C_2_ symmetry axis. Atomic color codes: Cl green, C grey, and H white.

To directly investigate the dynamics of chlorobenzene, we performed the analysis of the line shapes of static ^2^H quadrupole echo NMR spectra of **1**‐ClBz_d_5_ and **2**‐ClBz_d_5_ recorded at variable temperature (Figures 6, 7). Indeed, powder ^2^H NMR spectra, resulting from the superposition of the signals from all the possible orientations of C‐D bonds in the sample, are mainly determined by the quadrupolar interaction anisotropy, possibly reduced by molecular motions to give line shapes characteristic of the motion geometry and rate. In the powder spectrum of molecules rigid on the NMR time scale (i.e., undergoing motions with a rate < 10^4^ s^−1^), one can observe inner singularities split by $\delta_{0}\left(1-\eta_{0}\right)$, inner edges separated by $\delta_{0}\left(1+\eta_{0}\right)$, and a total width of 2$\delta_{0}$. $\delta_{0}$ and $\eta_{0}$ are the anisotropy and asymmetry parameters related to the ^2^H quadrupolar coupling constant ($C_{Q}=\frac{e^{2}qQ}{h}=eQV_{\mathrm{zz}}$) and field gradient on the nucleus, associated to a tensor with principal components ($V_{\mathrm{xx}},V_{\mathrm{yy}},V_{\mathrm{zz}})$ and *z* axis directed along the C‐D bond. In particular, $\delta_{0}=3/4C_{Q}$ and $\eta_{0}=\left(V_{\mathrm{yy}}-V_{\mathrm{xx}}\right)/V_{\mathrm{zz}}$. When $\eta_{0}$ is negligible, inner singularities and edges converge, and the so‐called Pake spectrum is obtained. When C‐D bonds undergo a rapid motion (i.e., with a rate >10^6^ s^−1^), an “averaged” spectrum results with singularities corresponding to averaged δ and η parameters, which depend on the motion geometry. For instance, for the chlorobenzene phenyl ring flipping by 180° about its C_2_ symmetry axis (π‐ flip), deuterons in *ortho* and *meta* positions with respect to chlorine are subject to fast exchange between two equivalent sites forming with the C_2_ axis an angle *θ* of about 60°, whereas the deuteron in the *para* position is not affected by the motion. Therefore, for *ortho* and *meta* deuterons, considering $\eta_{0}=0$, a spectrum with singularities at $\pm 5\delta_{0}/8,$ $\mp \delta_{0}/8$ and $\mp \delta_{0}$ is expected. A Pake spectrum with singularities split by $\delta_{0}$ and $2\delta_{0}$ is instead expected for the *para* deuteron.^[^ ^37^, ^66^ ^]^ In the case of fast isotropic reorientations, the quadrupolar interaction is averaged to zero and the powder spectrum collapses into a Lorentzian line.^[^ ^67^, ^68^ ^]^

All this considered, the spectra of **1**‐ClBz_d_5_ recorded at −70, −35, and 20 °C (Figure 6) can be interpreted as the superposition of a sharp and intense isotropic signal arising from chlorobenzene molecules undergoing fast random motions in the CP framework and a broad, less intense signal arising from chlorobenzene molecules undergoing restricted anisotropic dynamics. In fact, the latter signal can be decomposed into a Pake spectrum (*para* deuteron) and an averaged spectrum resulting from the fast π‐flip of the chlorobenzene molecules (*ortho* and *meta* deuterons) about their C_2_ symmetry axis, the two sub‐spectra being in a 1:4 intensity ratio.

As shown in Figure 6, the experimental spectra were well reproduced by the combination of a Lorentzian function (line width of 4 kHz), which represents the isotropic signal, and a line shape representing chlorobenzene molecules undergoing fast π‐flip calculated considering *δ*_0_ = 126 kHz, *η*_0_ = 0.08, *θ* = 57°, and a line width of 7 kHz. The isotropic signal accounts for 65–68% of the whole intensity in the investigated temperature range (Table S5).

The ^2^H spectra recorded on **2**‐ClBz_d_5_ from −70 to 20 °C show features of both a Pake pattern and a powder spectrum resulting from the fast π‐flip of the chlorobenzene phenyl ring about the C_2_ symmetry axis (Figure 7). Indeed, a good reproduction of the experimental spectra was obtained at all temperatures by the combination of two sub‐spectra with distinct line shapes, shown in blue and red in Figure 7. Discrepancies are mainly observed for the outer spectral edges, which are smoothed in the experimental spectrum due to finite pulse width.^[^ ^69^ ^]^ The sub‐spectrum in blue arises from ^2^H nuclei in *ortho* and *meta* positions with respect to the chlorine atom of chlorobenzene molecules undergoing fast π‐flip. The other sub‐spectrum, shown in red, is a Pake pattern that is given by ^2^H nuclei that lie on the *para*‐axis of molecules undergoing fast π‐flip, as well as ^2^H nuclei belonging to rigid chlorobenzene molecules. In fact, the ratio between the integral intensities of the two sub‐spectra is much less than 4:1, as expected if all molecules underwent fast π‐flip, and it decreases with decreasing temperature. Both sub‐spectra were calculated using *δ*_0_ = 126 kHz and *η*_0_ = 0.08. For the π‐flip, a Gaussian distribution of angles centered at *θ* = 57° with a full width (2σ) of 2–5°, increasing by decreasing the temperature, was used, with a line width of 5 kHz. The Pake pattern was calculated with a linewidth of 10 kHz. At each temperature, the fraction, *F*, of chlorobenzene molecules undergoing fast π‐flip was calculated from the simulation (Table 3).

**Table 3:** Integral intensities (%) of ^2^H sub‐spectra with fast π‐flip and Pake line shapes obtained from the analysis of ^2^H static spectra of **2**‐ClBz_d_5_, and fraction (*F*) of mobile chlorobenzene molecules.

T [°C]	Fast π‐flip [%]	Pake [%]	*F*
−70	36	64	0.45
−50	42	58	0.53
−35	47	53	0.59
−20	48	52	0.60
0	55	45	0.69
20	62	38	0.77

The evolution with temperature of the ^2^H NMR spectra of chlorobenzene‐d_5_ in **1**‐ClBz_d_5_ and **2**‐ClBz_d_5_ is typical of an “apparent phase transition” protracted over a broad temperature range. This behavior is generally due to a broad distribution of correlation times for the reorientational motion of the guest molecule, in turn determined by a temperature‐independent distribution of activation energies associated with different local microenvironments.^[^ ^70^, ^71^, ^72^, ^73^ ^]^ In our systems, the activation energy distribution could arise from different intermolecular distances and orientations in chlorobenzene‐framework interactions.

For chlorobenzene‐d_5_ in **2**‐ClBz_d_5_, the activation energy of the π‐flip motion can be determined from the population of mobile molecules, *F*, obtained from the line shape analysis (Table 3).^[^ ^74^, ^75^ ^]^ To this aim, the William‐Watts model^[^ ^76^ ^]^ was adopted to take into account the distribution of correlation times through the correlation function:

(9) $$g\left(t\right)\propto e^{-{\left(\frac{t}{\tau_{p}}\right)}^{\alpha }}$$

where *α* is a parameter describing the breadth of the distribution (0≤α≤1, with α = 0 corresponding to a flat distribution and α = 1 to a single correlation time) and $\tau_{p}$ indicates the distribution position on the time axis. For very broad distributions,^[^ ^75^ ^]^ in which only the fast and rigid limits are discernible, as in the ^2^H spectra of **2**‐ClBz_d_5_ (Figure 7), α≤0.3. In these conditions, considering an Arrhenius dependence of $\tau_{p}$ on temperature, the following equation holds true for the fraction, *F*, of molecules undergoing π‐flip in the fast limit:

(10) $$\;\ln\left[\ln{\left(1-F\right)}^{-1}\right]=A-\alpha \frac{E_{a}}{RT}$$

where A depends on motion parameters, and $E_{a}$ is the activation energy of the motion. In Figure S9, the experimental values of $\ln[\ln{\left(1-F\right)}^{-1}]$ are reported against 1000/T; a linear trend is observed, which can be fitted to Equation 10 to estimate the activation energy for the π‐flip motion. From the slope of the linear fitting, $\alpha E_{a}$= 4.7 kJ/mol is obtained; under the assumption of a broad correlation time distribution (α≤0.3), we can infer that $E_{a}$≥16 kJ/mol. This estimation is compatible with the values of the conversion factor between energy and absolute temperature scale determined by Rössler and coworkers from the analysis of ^2^H static spectra of benzene and hexamethylbenzene molecules in polymeric matrices.^[^ ^70^, ^71^ ^]^ Indeed, activation energies on the order of 27–40 kJ/mol correspond to the investigated temperature range.

In the case of **1**‐ClBz_d_5_, the slight change with temperature of the fraction of chlorobenzene molecules in the fast regime, corresponding to the isotropic sub‐spectrum, could arise from a nonperfect refocusing of the isotropic signal by the quadrupole echo; this hampers the estimation of the activation energy for the chlorobenzene motion in this sample.

As for the different geometry of the motion of chlorobenzene molecules in **1**‐ClBz_d_5_ (mainly isotropic reorientation) with respect to **2**‐ClBz_d_5_ (π‐flip motion about the C_2_ axis), this can be accounted for by considering the space available within the pockets between bispidine arms in the two CPs. The crystallographic analysis shows that, in the case of **2**‐ClBz, the ideal spherical volume that fills the space occupied by the two crystallographically independent chlorobenzene molecules, taken individually, is about 92 and 113 Å^3^,^1^ to be compared with the chlorobenzene volume of 97 Å^3^, as evaluated by considering the Van der Waals radii. Instead, in the case of **1**‐ClBz, an internal volume of about 137 Å^3^ is determined for the pocket hosting the chlorobenzene molecule. It is therefore evident that in **1**‐ClBz each chlorobenzene molecule has enough space within the pockets to move without orientational restraints, whereas in **2**‐ClBz the more limited available space and the chlorobenzene‐chlorobenzene interactions within the pockets only allow orientationally restricted motions.

## 4 Conclusion

High‐ and low‐resolution SSNMR experiments were combined to obtain information on structural and dynamic properties of host framework and guest molecules (chlorobenzene) in Hg CPs with different topologies, namely zig‐zag (**1**‐ClBz) and polycatenated (**2**‐ClBz).

The acquired data point to quite different dynamics of chlorobenzene molecules when trapped in the two CPs. The first evidence comes from the signal of chlorobenzene protons in ^1^H DE/MAS spectra, which is much narrower for **1**‐ClBz compared to **2**‐ClBz, indicating a higher degree of mobility of chlorobenzene molecules in the former. Analyses of ^1^H FIDs and ^2^H spectral line shapes on samples entrapping either chlorobenzene or chlorobenzene‐d_5_ confirmed and more quantitatively described the differences highlighted by ^1^H MAS spectra. It was found that in **1**‐ClBz chlorobenzene molecules show a liquid‐like behavior, while in **2**‐ClBz they exhibit restricted dynamics (π‐flip motion), with frequencies ranging from the rigid to the fast limit, with the fraction of more mobile molecules increasing with temperature. The different dynamics of the adsorbate in the two CPs is most probably associated with the different available volumes of the pockets hosting the solvent in the CP frameworks. In both cases, a broad distribution of activation energies was found for the chlorobenzene motion arising from the distribution of intermolecular distances and orientations in the guest‐host interactions.

As far as the dynamics of the ligands in the two CPs are concerned, ^1^H FID analysis indicated quite restricted motions in both cases. For **1**‐ClBz, the analysis of ^1^H and ^13^C spin‐lattice relaxation times versus temperature pointed out that the only activated motions in the fast MHz regime are the reorientations of the methyl groups around their C_3_ symmetry axes. Among them, the slower motion (reorientation of the NCH_3_ methyl group) is the relaxation sink of ^1^H nuclei. For **2**‐ClBz, the ^1^H *T*_1_ values and their trend with temperature, very similar to those observed for 1‐ClBz, suggest that the dynamics of the ligand are only slightly affected by the topology of the CP.

## Supporting Information

Supporting Information contains: P‐XRD patterns; solvent content evaluation by ^1^H NMR analysis in solution; ^13^C CP/MAS NMR spectra; ^1^H CPMG measurements; ^1^H and ^13^C longitudinal relaxation times; fractions of rigid and mobile chlorobenzene molecules in 1‐ClBz and 2‐ClBz from ^2^H NMR spectra.

## Author Contribution

The manuscript was written through the contributions of all authors. All authors have given approval to the final version of the manuscript.

## Conflict of Interest

The authors declare no conflict of interest.

## Supporting information



Supporting Information

## Data Availability

The data that support the findings of this study are available from the corresponding author upon reasonable request.

## Footnotes

1 The calculation of the volume hosting the chlorobenzene molecules was performed *in silico* using the Discovery Studio Visualizer program. Specifically, an ideal sphere was generated at the center of the chlorobenzene molecule and was gradually expanded until it filled the empty space defined by the surrounding CP fragments. The volume of the spheres was calculated and compared in the two crystal structures.
