# Supplementary material for: Dynamics of Ligand and Guest in 1D Hg(II)‐Bispidine Coordination Polymers With Different Topologies Investigated by Solid‐State NMR
Source: Chemistry. 2025 Jul 14;31(43):e202501458. doi: 10.1002/chem.202501458 (PMC12319395; doi:10.1002/chem.202501458)
Supplement: Supplementary file 1 — Supporting Information [file CHEM-31-e202501458-s001.pdf]

Supporting Information

Dynamics of Ligand and Guest in 1D Hg(II)-Bispidine Coordination Polymers with Different Topologies Investigated by Solid-State NMR

Elisa Della Latta,#[a] Francesco Della Croce,#[b] Giuditta Bizai,[a] Andrea Murelli,[c] Martina Lippi,[d] Patrizia Rossi,[d] Massimo Cametti,[c] Paola Paoli,[d] Francesca Martini,*,[a,b,e] Lucia Calucci,*,[b,e] Marco Geppi[a,b,e]

[a] Dr. E. Della Latta, BSc G. Bizai, Dr. F. Martini, Prof. M. Geppi
Dipartimento di Chimica e Chimica Industriale
Università di Pisa
via G. Moruzzi 13, 56124 Pisa, Italy
E-mail: francesca.martini@unipi.it

[b] MSc F. Della Croce, Dr. F. Martini, Dr. L. Calucci, Prof. M. Geppi
Istituto di Chimica die Composti OrganoMetallici – ICCOM
Consiglio Nazionale delle Ricerche – CNR
via G. Moruzzi 1, 56124 Pisa, Italy
E-mail: lucia.calucci@cnr.it

[c] MSc A. Murelli, Prof. M. Cametti
Dipartimento di Chimica, Materiali e Ingegneria Chimica "Giulio Natta"
Politecnico di Milano
Via Luigi Mancinelli, 7, 20133 Milano, Italy

[d] Dr. M. Lippi, Prof. P. Paoli
Dipartimento di Ingegneria Industriale
Università degli Studi di Firenze
via S. Marta 3, 50136 Firenze, Italy

[e] Dr. F. Martini, Dr. L. Calucci, Prof. M. Geppi
Centro per la Condivisione della Strumentazione Scientifica dell'Università di Pisa – CISUP
Lungarno Pacinotti 43/44, 56126 Pisa, Italy

S1. Structural characterization of CPs

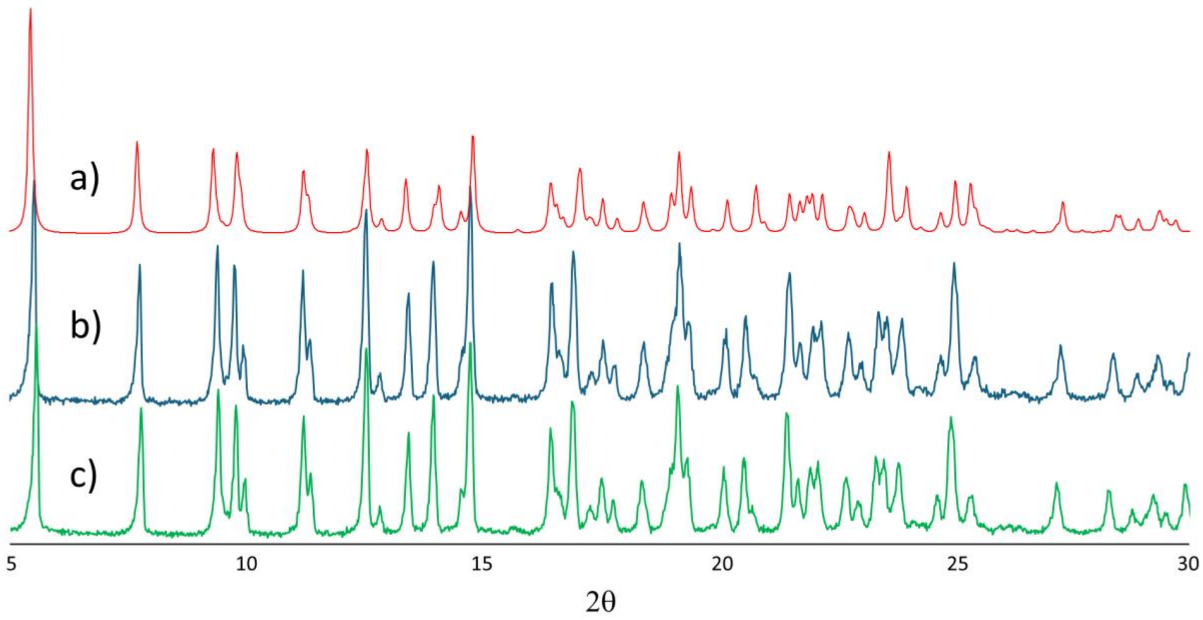

Figure S1. P-XRD simulated pattern for **1**-ClBz (a) and experimental P-XRD patterns for **1**-ClBz (b) and **1**-ClBz_d_5 (c), showing correspondence between the SC and microcrystalline powder phases.

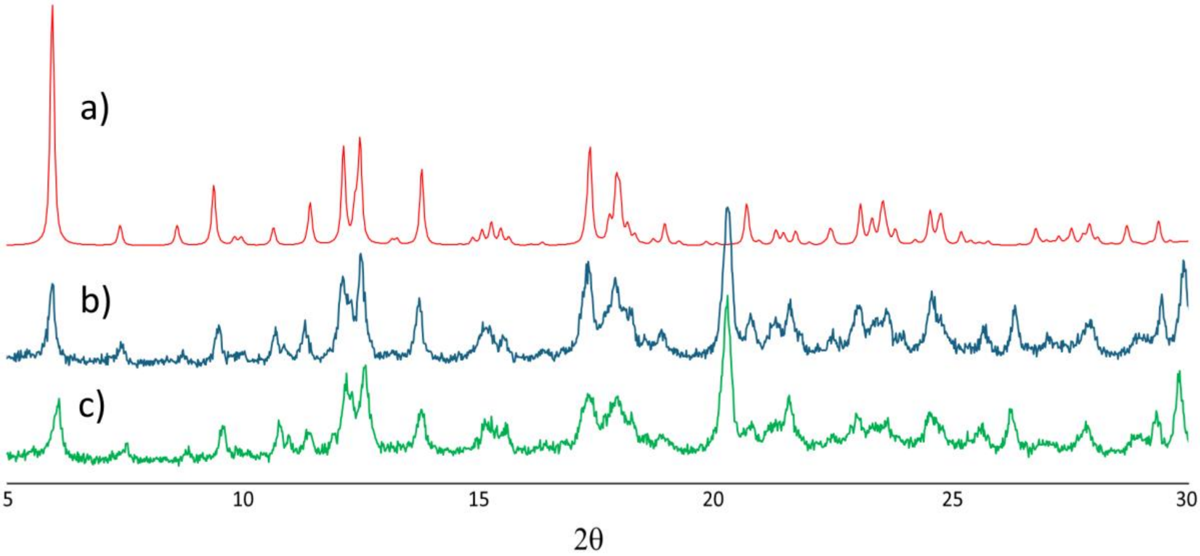

Figure S2. P-XRD simulated pattern for **2**-ClBz (a) and experimental P-XRD patterns for **2**-ClBz (b) and **2**-ClBz_d_5 (c), showing correspondence between the SC and microcrystalline powder phases.

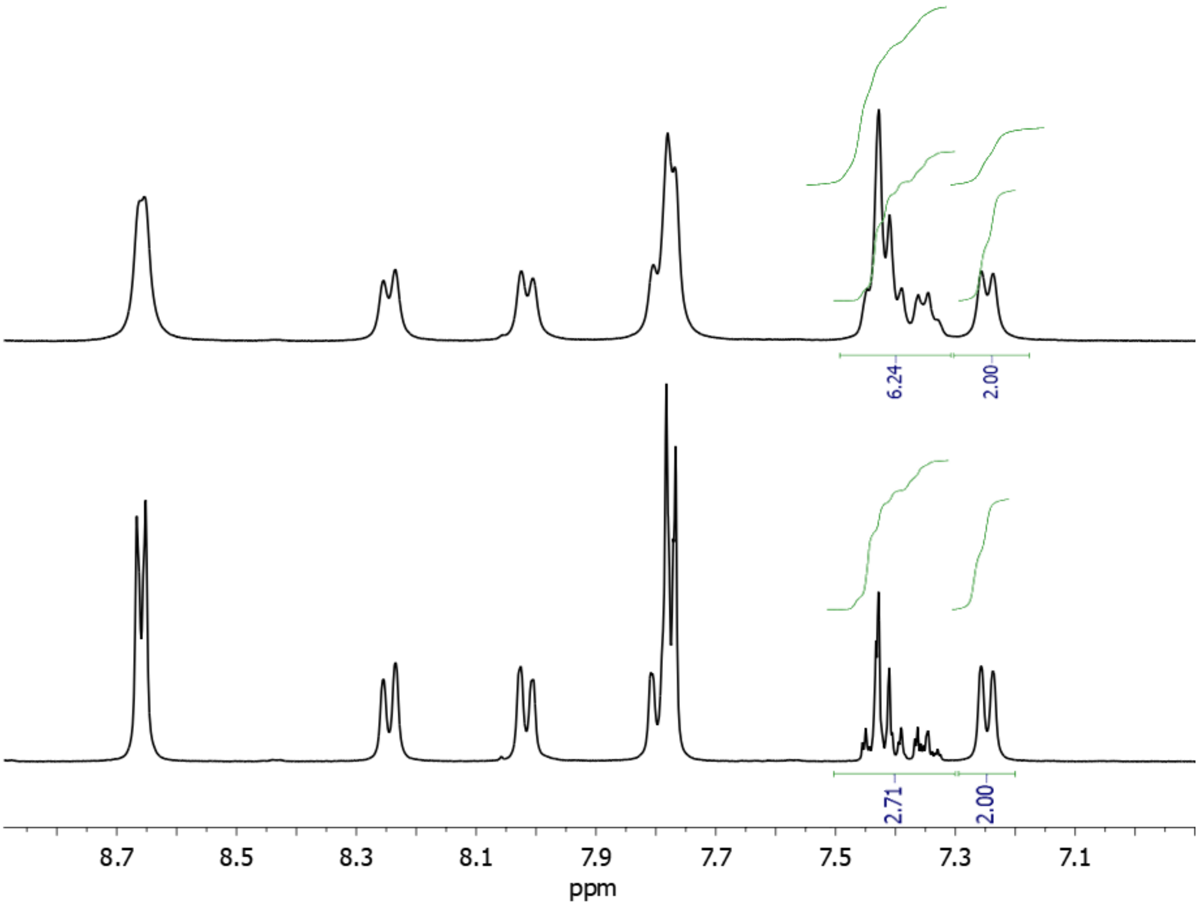

Figure S3. Portions of ^1H NMR spectra (400 MHz, dmso-d_6, 298 K) of as-synthesized CPs with evaluation by integral analysis of the chlorobenzene content: (bottom) **1**-ClBz = 0.54 chlorobenzene molecules per ligand and (top) **2**-ClBz = 1.25 chlorobenzene molecules per ligand.

P-XRD confirms the structural integrity of both CPs. The small discrepancy between the chlorobenzene content determined by SC-XRD (0.5 and 1.5 molecules per ligand for **1**-ClBz and **2**-ClBz, respectively) and that estimated by solution-state ^1H NMR spectroscopy on digested microcrystalline powders (0.54 and 1.25 molecules per ligand for **1**-ClBz and **2**-ClBz, respectively) is ascribable to several factors. On one hand, SC-XRD provides an idealized model based on a selected single crystal measured under cryogenic and sealed conditions, which does not account for potential guest loss in bulk material. On the other hand, the NMR-based quantification, while very useful in the case of CPs with non-paramagnetic metals, is inherently less precise due to integration errors. Moreover, minor sample variation and partial solvent loss during isolation, handling, and storage of the microcrystalline samples may contribute to variations in chlorobenzene content in the bulk powders. Finally, the presence of small impurities or amorphous content cannot be excluded entirely, as microcrystalline powders rarely reflect 100 % purity. All this considered, the chlorobenzene content determined by ^1H NMR remains within a range sufficient for the comparative dynamic analysis performed by SSNMR.

S2. High-resolution SSNMR spectra

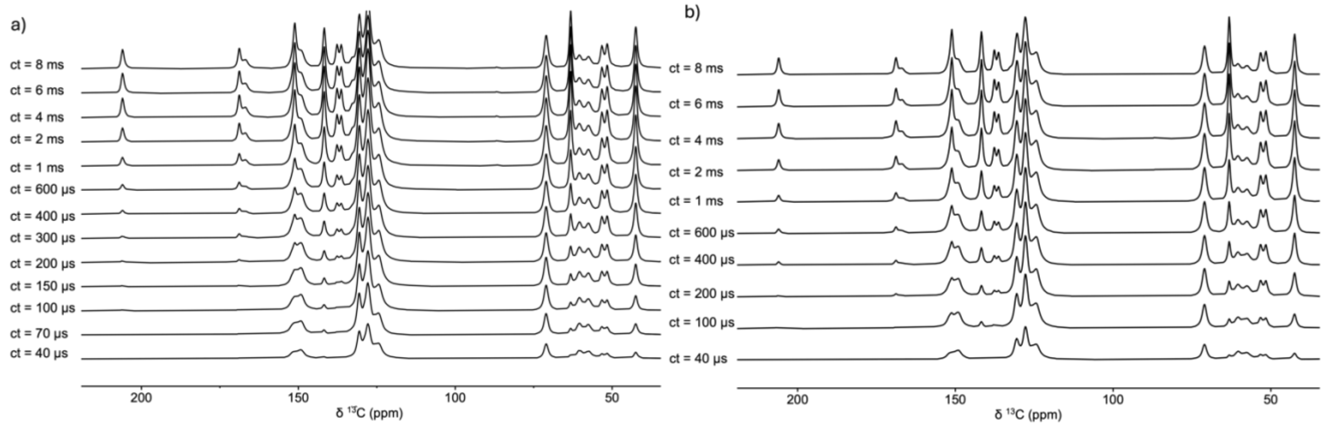

Figure S4. ^{1}H-^{13}C CP/MAS spectra of **1**-ClBz (a) and **1**-ClBz_d$_5$ (b) recorded with the indicated contact time (ct) values. The spectra were recorded at 25 °C using a spinning frequency of 15 kHz.

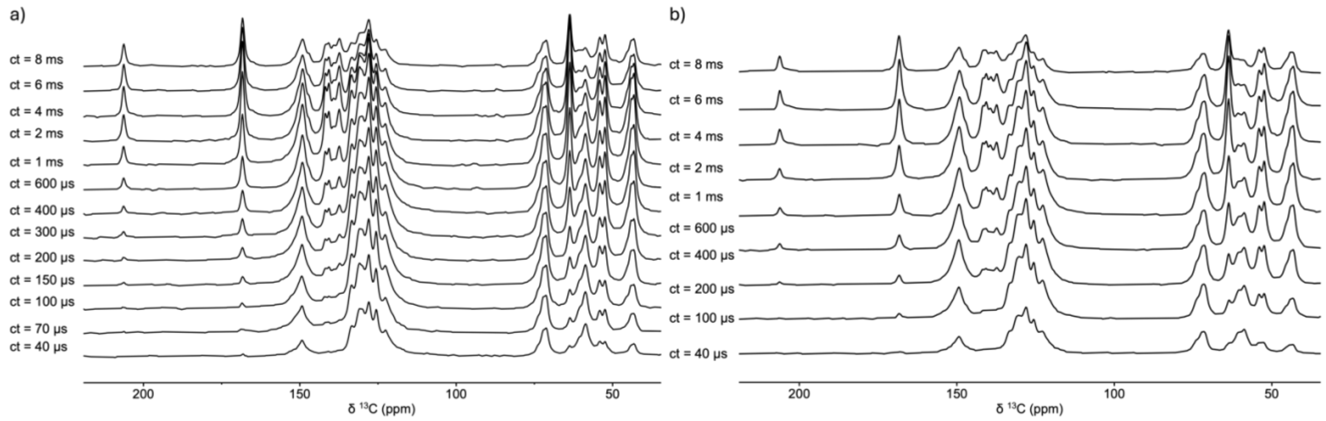

Figure S5. ^{1}H-^{13}C CP/MAS spectra of **2**-ClBz (a) and **2**-ClBz_d$_5$ (b) recorded with the indicated contact time (ct) values. The spectra were recorded at 25 °C using a spinning frequency of 15 kHz.

S3. ^{1}H T_2 measurements

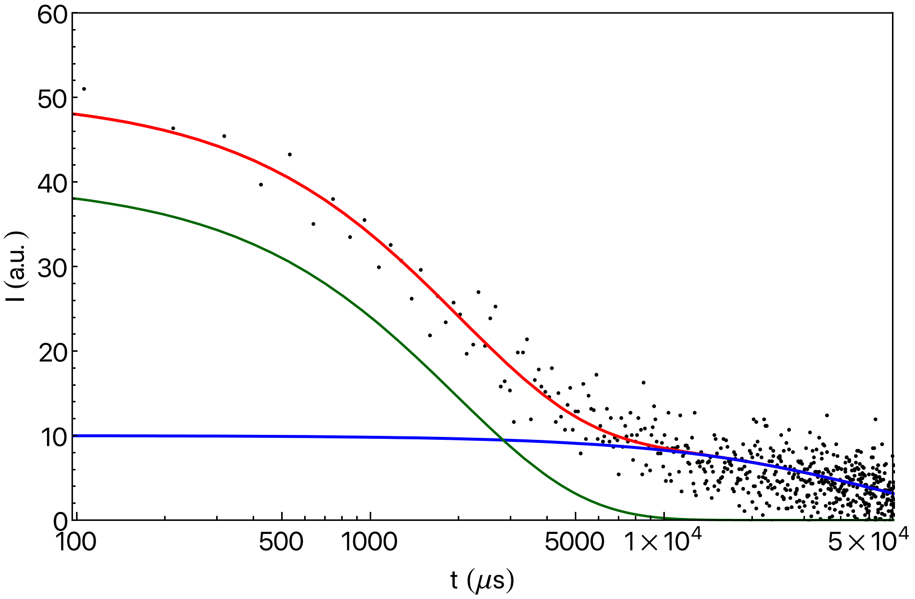

Figure S6. ^{1}H CPMG decay recorded on sample **1**-ClBz at 20 °C. Black dots are the experimental data, red line is the fitting function, and blue and green lines are the single exponential components.

S4. ^{1}H and ^{13}C T_1 analysis

Table S1. ^{1}H T_1 values determined at 20.8 MHz for **1**-ClBz, **1**-ClBz_d$_5$, **2**-ClBz, and **2**-ClBz_d$_5$.

T (°C)	T_1 (s)			
	**1**-ClBz	**1**-ClBz_d$_5$	**2**-ClBz	**2**-ClBz_d$_5$
20	0.89	0.80	0.68	0.74
0	0.69	0.62	0.70	0.54
10	0.81	0.74	0.73	0.70
–10	0.60	0.52	0.61	0.42
–20	0.49	0.48	0.49	0.29
–30	0.39	0.35	0.37	0.22
–40	0.32	0.26	0.31	0.15
–50	0.25	0.20	0.23	0.13
–60	0.20	0.16	0.18	0.12
–70	0.16	0.13	0.17	0.12
–80	0.14	0.13	0.17	0.13
–90	0.14	0.14	0.19	0.15
–100	0.14	0.15	0.22	0.15

Table S2. ^{13}C T_1 values determined for 1-ClBz at 125.77 MHz. The numbering refers to the ligand in Scheme 1 and to the spectral assignment in Figure 3.

T (°C)	T_1 (s)													
	**C1/C17**	**C2**	**C3**	**C4**	**C5**	**C6**	**C7**	**C8**	**C9,C13**	**C10,C12**	**C11**	**C14**	**C15**	**C16**
30	0.64						8.9							
40	0.79						10.8							
51	0.84	>40	>60	>35	>50	>65	9.6	>70	>60	>30	>50	>70	>80	>50
62	1.1						13.4							
72	1.2						12.7							

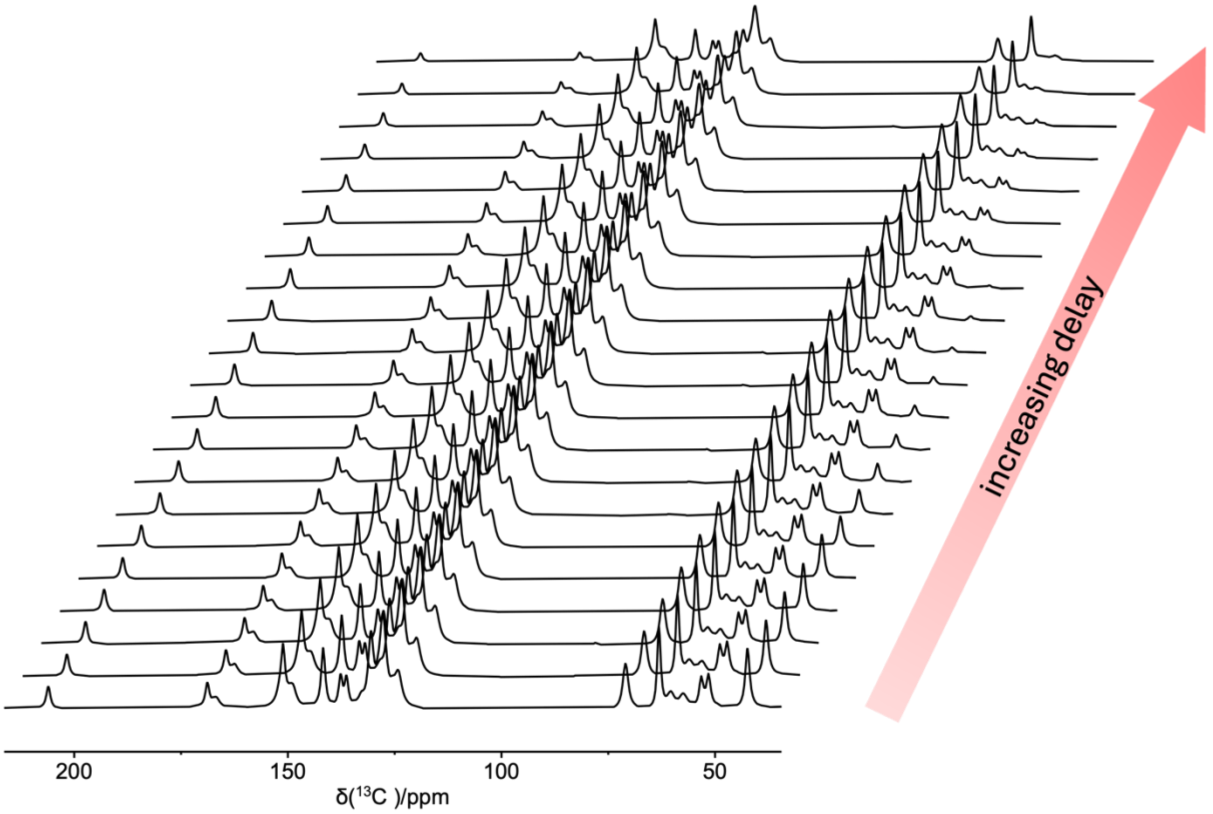

Figure S7. ^{13}C Torchia experiment recorded on **1**-ClBz at 51 °C.

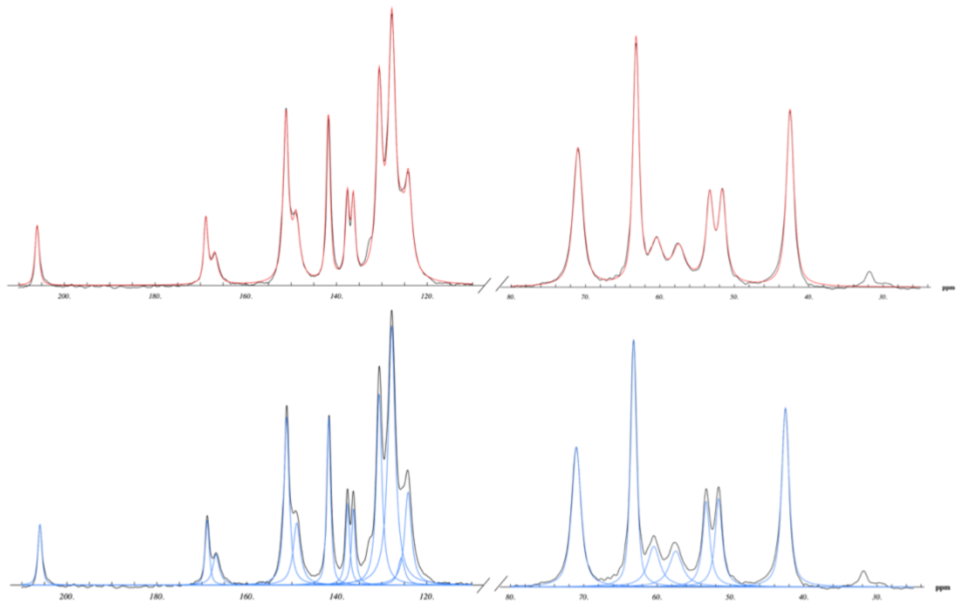

Figure S8. Example of deconvolution of the ^{13}C spectra obtained from the Torchia experiment (51 °C with a recovery delay of 10 ms). The experimental spectrum is in black, while the simulated spectrum is reported in red and the single signals in blue.

Table S3. Best fit parameters obtained from the global fitting of the ^1H and ^{13}C T_1 curves *vs* temperature (Figure 5) of **1**-ClBz in terms of Equations 3-8.

Parameter	NCH_3	$COOCH_3$
**E_a (kJ/mol)**	9.1	8.4
**log (τ_∞)**	-12	-13
**τ_c (ps) at 25 °C**	23	2.4
**K_H (s^{-2})**	$4.1 \cdot 10^5$	$4.1 \cdot 10^5$

^{1}H longitudinal relaxation

Considering that the relaxation sink of ^1H nuclei is the reorientation of the NCH_3 methyl groups, the measured relaxation rate can be expressed as:

$$\langle R_1 \rangle = \langle \frac{1}{T_1} \rangle = \frac{f^{NCH_3}}{T_{1NCH_3}}$$

where f^{NCH_3} and T_{1NCH_3} are the fractional weight and the T_1 value for the NCH_3 methyl protons, while a practically null relaxation rate (*i.e.*, $T_1 \rightarrow \infty$) is considered for all the other protons in the linker and adsorbate. Based on this expression, the average longitudinal relaxation time measured for the different samples is inversely proportional to f^{NCH_3}, or, in other words, directly proportional to the number of hydrogens in the sample, if T_{1NCH_3} is considered equal for all samples. Considering the sample formula and chlorobenzene deuteration, f^{NCH_3} is expected to be 0.16 in **1**-ClBz, 0.14 in **2**-ClBz and 0.18 in both **1**-ClBz_d$_5$ and **2**-ClBz_d$_5$. Therefore, values of about 0.89 and 0.78 are expected for the ratios between the measured T_1 values of **1**-ClBz_d$_5$ and **1**-ClBz and of **2**-ClBz_d$_5$ and **2**-ClBz, respectively. These values are in good agreement with the experimental values reported in Table S4, confirming that the motion of the NCH_3 methyl groups is indeed the relaxation sink for the investigated samples and that chlorobenzene protons do not contribute to relaxation at the investigated Larmor frequency.

Table S4. Values of the ^1H T_1 ratios for the indicated samples at different temperatures

T (°C)	$\frac{T_1(1-\mathrm{ClBz\_d_5})}{T_1(1-\mathrm{ClBz})}$	$\frac{T_1(2-\mathrm{ClBz\_d_5})}{T_1(2-\mathrm{ClBz})}$
20	0.9	1.1
0	0.9	0.8
10	0.9	1.0
–10	0.9	0.7
–20	1.0	0.6
–30	0.9	0.6
–40	0.8	0.5
–50	0.8	0.6
–60	0.8	0.7
–70	0.8	0.7
–80	0.9	0.8
–90	1.0	0.8
–100	1.1	0.7

S5. ^{2}H NMR

Table S5. Percentage of the isotropic and anisotropic (π-flip) components in the simulated ^{2}H quadrupole echo NMR spectra of **1**-ClBz_d_5 at the indicated temperatures.

T (°C)	% isotropic	% π-flip
−70	65	35
−35	68	32
20	68	32

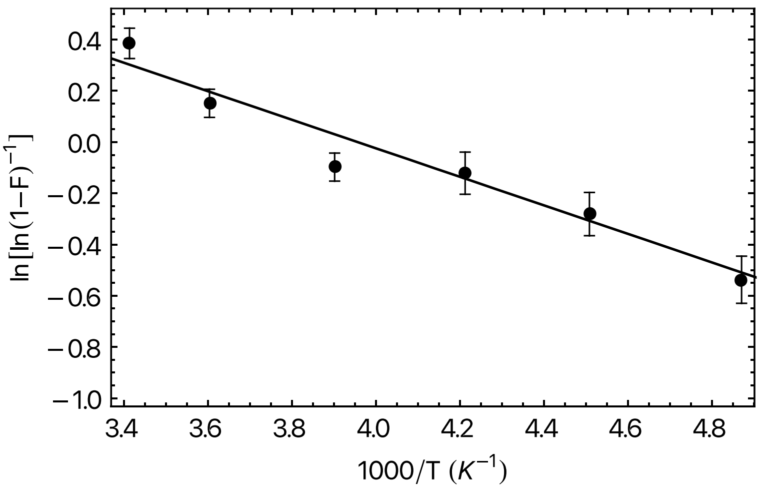

Figure S9. ln[ln(1 − F)$^{-1}$] *vs* 1000/T for **2**-ClBz_d_5. The black line corresponds to the linear fitting in terms of Equation 10.
